# Culturing Conditions Dictate the Composition and Pathways Enrichment of Human and Rat Perirenal Adipose-Derived Stromal Cells’ Secretomes

**DOI:** 10.1007/s12015-024-10748-w

**Published:** 2024-06-26

**Authors:** Erika Pinheiro-Machado, Marijke M. Faas, Bart J. de Haan, Cyril Moers, Alexandra M. Smink

**Affiliations:** 1 Department of Pathology and Medical Biology, University of Groningen, University Medical Center Groningen, Hanzeplein 1 (EA11), Groningen, 9713 GZ The Netherlands; 2 Department of Surgery – Organ Donation and Transplantation, University of Groningen, University Medical Center Groningen, Groningen, The Netherlands

**Keywords:** Adipose-derived stromal cells, Secretome, Culturing conditions, Enrichment analysis, Regenerative medicine, Secretomics

## Abstract

Understanding the impact of various culturing strategies on the secretome composition of adipose-derived stromal cells (ASC) enhances their therapeutic potential. This study investigated changes in the secretome of perirenal ASC (prASC) under different conditions: normoxia, cytokine exposure, high glucose, hypoxia, and hypoxia with high glucose. Using mass spectrometry and enrichment clustering analysis, we found that normoxia enriched pathways related to extracellular matrix (ECM) organization, platelet degranulation, and insulin-like growth factor (IGF) transport and uptake. Cytokine exposure influenced metabolism, vascular development, and protein processing pathways. High glucose affected the immune system, metabolic processes, and IGF transport and uptake. Hypoxia impacted immune and metabolic processes and protein processing. Combined hypoxia and high glucose influenced the immune system, IGF transport and uptake, and ECM organization. Our findings highlight the potential of manipulating culturing conditions to produce secretomes with distinct protein and functional profiles, tailoring therapeutic strategies accordingly.

**Graphical Abstract:**

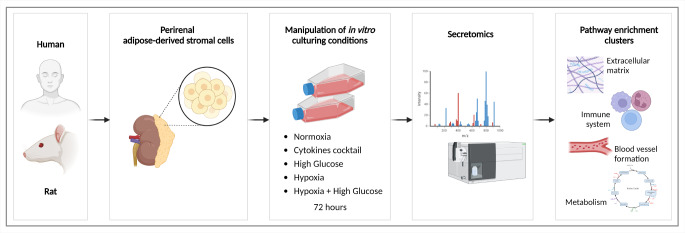

**Supplementary Information:**

The online version contains supplementary material available at 10.1007/s12015-024-10748-w.

## Introduction

Adipose-derived stromal cells (ASC) play a pivotal role in regenerative medicine, showing promise for treating diverse conditions such as cardiovascular disorders, bone regeneration, systemic lupus erythematosus, and multiple sclerosis [1–6]. ASC exhibit immunomodulatory [7–10], proangiogenic [11–14], remodeling, and repairing abilities [14, 15]. Additionally, they have minimal immunogenicity [16], presenting fewer ethical and technical concerns compared to embryonic stem cells. The mechanisms behind such activities of ASC are not yet fully described, but they are mainly thought to act via the secretion of autocrine and paracrine factors [13]. These include various growth factors [13], extracellular matrix (ECM) proteins, RNA, and exosomes [15, 17], collectively known as the ASC secretome.

In the past decade, extensive research has demonstrated that the ASC secretome contains regeneration-promoting factors facilitating tissue and organ repair [18–20]. Therefore, next to ASC-based cell therapy, an ASC cell-free strategy using only the ASC secretome may be a safe and promising tool for various medical purposes.

One of the advantages of such an ASC cell-free strategy is that the ASC secretome composition can be modulated in vitro. The ASC secretome is not a predefined and immutable mixture. Many factors influence its composition [13], and biochemical stimuli (e.g., exposure to specific culturing conditions) are interesting in vitro strategies known to modulate ASC secretion [12, 21–23]. Much of the research about the ASC secretome revolves around different culturing strategies that stimulate ASC to release a different set of molecules [21, 23, 24]. The main idea is that certain molecules of interest may be enriched in the ASC secretome upon culturing. Such enrichment may generate a mixture capable of stimulating different physiological processes such as vascularization, immunomodulation, and ECM remodeling [15, 18, 25–27].

The standard in vitro culturing condition is normoxic culturing (21% O_2_); ASC cultured under normoxic conditions secrete molecules such as basic fibroblast growth factor (bFGF), hepatocyte growth factor (HGF), vascular endothelial growth factor (VEGF), and various cytokines [13, 23, 28]. Exposure to proinflammatory factors, for instance, interferon-gamma (IFN-^y^), tumor necrosis factor-alpha (TNF-α), interleukin-1β (IL-1β), or lipopolysaccharides (LPS), has been used to induce the release of proinflammatory cytokines such as IL-1β, IL-6, IL-8, IL-12, IFNs, and TNF-α into the secretome [26, 29, 30]. . Hypoxia has been used to increase the secretion of proangiogenic factors, such as VEGF, HGF, and FGF-2 [12, 17]. Other culturing strategies mimicking microenvironments of interest (i.e., high or low glucose, a combination of multiple stimuli) may reveal new ASC secretome mixtures that play a role in health and disease.

This comprehensive study describes the isolation, culture, and characterization of human (h-prASC) and rat perirenal ASC (r-prASC). Next, we investigated the perirenal ASC secretome composition changes upon the most employed culturing strategies used to mimic physiological and non-physiological conditions, i.e., cytokine exposure; exposure to high glucose; exposure to hypoxia; and exposure to a combination of hypoxia and high glucose, which generated a comprehensive database using an unbiased proteomic approach by collecting culturing-dependent secretomes from both cell types. Qualitative and quantitative overviews of the secretomes are provided. Finally, *in-silico* functional analysis of the different secretomes reveals the relevant biological processes and pathways that are upregulated upon the different stimuli. With these data, we aim to support further prASC secretome investigations and contribute to their translational use.

## Materials and Methods

### Medium Preparation

This research used multiple medium types: 1: standard medium without serum (STD-M (-)), which is used for adipose tissue digestion and for both human and rat prASC culturing upon normoxia, exposure to cytokines, and hypoxia, and it contains Dulbecco’s Modified Eagles Medium 4.5 g/L D-glucose (DMEM; Lonza, Walkersville, MD, USA) supplemented with 50 U/mL penicillin, 50 mg/mL streptomycin (Corning Cellgro, Manassas, VA, USA) and 2 mM L-glutamine (Lonza); 2: standard medium with serum (STD-M (+)) which is used for both human and rat prASC isolation and expansion, and it contains DMEM 4.5 g/L D-glucose supplemented with 50 U/mL penicillin, 50 mg/mL streptomycin, 2 mM L-glutamine, and 10% heat-deactivated Fetal Bovine Serum (FBS; Thermo Fisher Scientific, Bleiswijk, The Netherlands); 3: adipogenic medium, which is used for both human and rat prASC differentiation into adipocytes and it contains DMEM 4.5 g/L D-glucose, 0.1 µM dexamethasone (Sigma-Aldrich, Zwijndrecht, The Netherlands), 1 nM insulin (Thermo Fisher Scientific), 0.5 mM 3-Isobutyl-1-methylxanthine (IBMX; Sigma-Aldrich), and 10% FBS; 4: osteogenic medium, which is used for both human and rat prASC differentiation into osteoblasts and it contains DMEM 4.5 g/L D-glucose, 0.1 µM dexamethasone, 10 mM b-glycerophosphate (Sigma-Aldrich), and 0.05 mM ascorbic acid (Sigma-Aldrich); 5: myogenic medium, which is used for both human and rat prASC differentiation into smooth muscle cells and it contains DMEM 4.5 g/L D-glucose, 0.1% TGF-β1 (PeproTech, Hamburg, Germany), and 10% FBS; 6: high glucose medium (HG- M (-)), which is used for both human and rat prASC secretome harvesting upon culturing in high glucose and in high glucose + hypoxia, and it contains DMEM 35mM D-glucose, supplemented with 50 U/mL penicillin, 50 mg/mL streptomycin, and 2 mM L-glutamine; 7: cytokine medium (Cyto-M (-)),which is used for ASC secretome harvesting upon cytokine exposure and contains DMEM 4.5 g/L D-glucose, supplemented with 50 U/mL penicillin, 50 mg/mL streptomycin, 2 mM L-glutamine, 20 ng/mL IFN-γ (ImmunoTools, Friesoythe, Germany), 21.5 ng/mL TNF-α (ImmunoTools), and 10 ng/mL IL-1β (ImmunoTools). Cyto-M (-) used for culturing rat prASC contained the same cytokines’ concentrations but used rat cytokines (ImmunoTools).

### prASC Preparation

#### Collection of Adipose Tissue

Human perirenal adipose tissue was obtained from living kidney donors at the University Medical Center Groningen as a clinical waste product. Anonymously donated samples were obtained with informed consent as approved by the ethical board of the University Medical Center Groningen, following the guidelines for waste materials. Donors comprised males (43%) and females (57%). The average age was 57.6 ± 11.5 years. Adipose tissue was stored at 4 ^o^C and processed within 48 h (h) post-surgery. Rat perirenal adipose tissue was obtained from male Sprague-Dawley rats (Envigo, Horst, The Netherlands) of 7–9 weeks old with a body weight ranging between 250 and 280 g. The Dutch Central Committee on Animal Testing (CCD) and Animal Welfare Authority at the University of Groningen approved this collection of adipose tissue (AVD10500202115138).

#### Processing Adipose Tissue

Human (h-prASC) and rat (r-prASC) perirenal adipose-derived stromal cells were isolated from 3 different donors. The isolation procedure followed the same steps for both species. Briefly, the collected tissue was extensively washed with Hank Balanced Salt Solution (HBSS; Thermo Fisher Scientific) and cut into small pieces. The tissue pieces were digested with collagenase neutral protease blend 4 (0.5 mg/mL; Nordmark Biochemical, Uetersen, Germany) in STD-M (-) at 37 ^o^C during continuous agitation for 30 min (min). Centrifugation separated cells from residual fibrous material, adipocytes, and lipid content (700 x g, 7 min, RT). The resulting pellet containing prASC was resuspended in STD-M (+).

#### Cell Expansion

Cells were plated in T25 flasks for initial cell culture [Passage 0] at 37 ^o^C and 5% CO_2_. After 24 h, ASC adhered and were repeatedly washed with phosphate-buffered saline (PBS; Thermo Fisher Scientific) to remove debris and non-adherent cells. The medium (STD-M (+)) was replaced every three days. Cells reaching 80% confluence were passaged by trypsinization. Cells were expanded until passage three and used for experiments.

### Cell Characterization

#### Immunophenotyping

To determine the cell phenotype, h-prASC and r-prASC (passage 3, *n* = 3) were trypsinized, and the pellet was resuspended in PBS. Subsequently, cells were washed with 1.0 mL Flow Cytometry Buffer (PBS containing 0.05% FBS) by centrifuging at room temperature for 5 min at 500 x g. The supernatant was discarded, and the cells were incubated with antibodies at 4 °C for 10 min. The antibodies were chosen based on multiple reports describing human ASC phenotypes and considering the minimal criteria set by The International Society for Cellular Therapy [31]. The h-prASC were labeled with CD29-Brilliant Violet 510 (1:50) (BD Biosciences, Vianen, The Netherlands), CD73-APCVio770 (1:50) (Miltenyi Biotec, Leiden, The Netherlands), CD90-PE-Cy 5.5 (1:50) (Novus Biologicals, Oxon, England), CD31-Brilliant Violet 605 (BioLegend, Amsterdam, The Netherlands), CD45-Alexa Fluor 647 (1:11) (BioLegend), and HLA-DR-Brilliant Violet 421 (1:50) (BioLegend). For the r-prASC characterization, cells were labeled with CD29-Alexa Fluor 555 (1:50) (Bioss, Bio-Connect, Huissen, The Netherlands), CD73-Alexa Fluor 680 (1:50) (Bioss), CD90-FITC (1:50) (Bioss), CD31-APC (1:50) (Miltenyi Biotec), CD45-Brilliant Violet 421 (1:50) (BD Biosciences), and MHC Class II RT1Bu (HLA-DR)-PerCP (Novus Biologicals). Subsequently, cells were washed with Flow Cytometer Buffer and centrifuged twice, as described above. Cells were resuspended in Flow Cytometer Buffer and analyzed using a NovoCyte Quanteon Flow Cytometer (Agilent, Middelburg, The Netherlands). Cells were gated based on their forward and side scatter, and unlabeled cells were used to set the gates: in the unstained sample, gates were set so that 99% of the cells were negative. This gate was then copied to the stained sample. The NovoExpress Software (Agilent, version 1.5.6) was used for analysis.

#### Colony-Forming Unit (CFU) Assay

To determine the stemness potential, the CFU capacity of h-prASC and r-prASC was measured (passage 3, *n* = 3). Briefly, cells were seeded at a low density (100 cells/well) in 6-well plates and cultured in STD-M (+) for 14 days. The culture medium was refreshed twice a week. After 14 days, wells were washed twice with PBS and fixed with 2% Paraformaldehyde (PFA, Sigma-Aldrich) in PBS for 15 min. Cells were washed thrice with PBS and stained with 0.05% Crystal Violet (Sigma-Aldrich) for 30 min. After staining, cells were washed twice with tap water and air-dried. The number of colonies was determined by counting the number of colonies larger than 50 cells. The colony-forming potential (% CFU) was compared by calculating the amount of colonies/number of cells seeded) ×100.

### Differentiation Potential

To investigate the differentiation potential of h-prASC and r-prASC, cells (passage 3, *n* = 3) were differentiated towards adipocytes, osteoblasts, and smooth muscle cells. Briefly, cells were seeded in 24-well plates and cultured in STD-M (+). When confluence was reached, cells were washed twice with PBS and incubated with adipogenic, osteogenic, or myogenic medium for 14 days. The medium was refreshed every three days. After 14 days, cells were fixed with 2% PFA (in PBS) and stained for 30 min with Oil Red O (0.005%, in isopropanol) (Sigma-Aldrich) for adipogenic differentiation, Alizarin Red (0.005%, in PBS) (Sigma-Aldrich) for osteogenic differentiation, and Phalloidin-FITC (1:250, in DMSO) (Invitrogen, Thermo Fisher Scientific) in a DAPI solution (1:5000, in ultrapure water) (Thermo Fisher Scientific) for smooth muscle cell differentiation. The Oil Red O and Alizarin Red staining were evaluated with a light microscope (Leica DM2000 LED microscope with a Leica DFC 450 camera (Leica Microsystems B.V., Amsterdam, The Netherlands)). Phalloidin-FITC staining was evaluated with an immunofluorescence microscope (EVOS Cell Imaging System, Invitrogen).

### Secretome Harvesting

#### Culturing Strategies

h-prASC and r-prASC (passage 3, *n* = 3) were seeded in 6-well plates at a density of 50,000 cells/well (5208 cells/cm^2^) and cultured in STD-M (+). Upon reaching 80% confluence, cells were repeatedly and carefully washed with PBS, and the medium was refreshed with STD-M (-). After 24 h, wells were divided into six culturing groups: cells cultured in STD-M (-) for the normoxic (21% O_2_, 5% CO_2_) and hypoxic (1% O_2,_ 5% CO_2_) culturing), Cyto-M (-) (for culturing with cytokines (21% O_2_, 5% CO_2_), and HG-M (-) (for culturing in high glucose (21% O_2_, 5% CO_2_) and for culturing in high glucose under hypoxia (1% O_2,_ 5% CO_2_). Cells were cultured for 72 h.

### Secretome Collection

After 72 h, the medium (h-prASC and r-prASC secretomes) was harvested. Secretomes were centrifuged at 2500 x g for 10 min to remove dead cells, large apoptotic bodies, and debris. Samples from each culturing group were collected individually (for mass spectrometry; *n* = 3) or pooled (we pooled three times three samples for Luminex and ELISA). All samples were aliquoted and snap-frozen in liquid nitrogen. Secretomes were stored at − 80 °C until further processing.

### Descriptive Assays

#### Secretome Processing

Secretome samples were thawed on ice, and the proteins were concentrated by a 90 min centrifugation step (14,000 x g) using a 3-kDa cut-off spin filter (Amicon Ultra-4 Centrifugal filter unit; Merck Millipore, Burlington, MA, USA), resulting in a 40–50-fold more concentrated final product. Following standard procedures, protein concentration was determined using Bio-Rad Protein Assay (Bio-Rad, Milan, Italy). Concentrated secretomes presented a total protein concentration of 0.62 ± 0.3 µg/uL.

#### Discovery-Based Proteomics Analyses

Protein levels were determined with discovery-based proteomics (label-free quantification) for relative protein concentrations [32]. Briefly, in-gel digestion was performed on 30 µL of the provided secretomes using trypsin (300 ng sequencing grade modified trypsin V5111; Promega, Leiden, The Netherlands) after reduction with 10 mmol/L dithiothreitol and alkylation with 55 mmol/L iodoacetamide proteins [33]. Discovery mass spectrometric analyses were performed on a quadrupole orbitrap mass spectrometer equipped with a nano-electrospray ion source (Orbitrap Exploris 480; Thermo Scientific). Chromatographic separation of the peptides was performed by liquid chromatography (LC) on an Evosep system (Evosep One; Evosep, Odense, Denmark) using a nano-LC column (EV1137 Performance column 15 cm x 150 μm, 1.5 μm, Evosep; buffer A: 0.1% v/v formic acid, dissolved in milliQ-H2O, buffer B: 0.1% v/v formic acid, dissolved in acetonitrile). The digests were injected in the LC-MS with the equivalent of 1 µL starting material, and the peptides were separated using the 30SPD workflow (Evosep). The mass spectrometer was operated in positive ion mode and data-independent acquisition mode (DIA) using isolation windows of 16 m/z with a precursor mass range of 400–1000, switching the FAIMS between CV-45 V and − 60 V with three scheduled MS1 scans during each screening of the precursor mass range.

#### Mass Spectrometry Data Analysis

LC-MS raw data were processed with Spectronaut (version 17.1.221229; Biognosys Inc, Cambridge, United States) using the standard settings of the directDIA workflow except that quantification was performed on MS1, with a human or rat SwissProt database (www.uniprot.org, 20.350 entries for the human samples and 8.094 entries for the rat samples). Local normalization was applied for quantification, and the Q-value filtering was set to the classic setting without imputing.

#### Mass Spectrometry in Silico Analysis

To gain insights into functional changes that might occur when different culturing strategies are applied to human and rat prASC, we submitted the lists of proteins from each human and rat prASC culturing strategy to Metascape [34]. For each secretome, Metascape carried out an analysis of pathway enrichment clusters from many ontology sources. The most statistically significant term within a cluster is then chosen (by Metascape) for representation. We first submitted the list of proteins of the normoxic secretomes from both human and rat prASC to Metascape. We used a list that consisted of proteins that were common to all three donors investigated for this. Then, we submitted the secretomes resulting from different culturing conditions: we submitted those proteins uniquely present in the respective culturing condition compared with the normoxic culturing condition.

#### Quantification of Paracrine Factors

To confirm the presence and quantify key paracrine factors associated with angiogenesis (basic fibroblast growth factor (bFGF), platelet-derived growth factor AB (PDGF), and vascular endothelial factor (VEGF)), extracellular matrix (collagen I alpha I), as well as immunomodulation (IL-10, and IL-1α), ELISA or Luminex was performed. The levels of rat and human bFGF, PDGF, VEGF (DuoSet ELISA, R&D Systems), and rat collagen I alpha I (Novus Biologicals, Biotechne, Minneapolis, USA) were measured using ELISA. Rat and human IL-10, IL-1α, and human collagen I alpha I were analyzed using magnetic Luminex® Assays (R&D systems; #LXSARM-03 / #LXSAHM-04). Samples were measured in triplicates. Assays were performed according to each manufacturer’s protocol. Briefly, for the magnetic Luminex®, the antibody magnetic bead mix was added to 96-well plates. The standards were resuspended, and serial dilutions were prepared. Standards and samples were added and incubated overnight at 4 °C. After washing, detection antibodies were added, and the plates were incubated for 30 min at room temperature while shaking. After incubation, the plates were rewashed and incubated with streptavidin-PE for 30 min at room temperature while shaking. Finally, the plates were rewashed, and 100 µl of wash buffer was added to each well. The plates were analyzed using a Luminex 200 System. The data obtained were analyzed using the Luminex xPONENT software. The manufacturer’s protocol was followed for the ELISA kits from both R&D systems and Novus Biologicals, and results were obtained using a microplate spectrophotometer Benchmark Plus BIO-RAD at 450 nm with correction at 540 nm.

#### Conventional Statistical Analysis

The experimental groups (i.e., cytokines, high glucose, hypoxia, and hypoxia + high glucose) were compared to the standard condition (normoxic secretome) for comparisons. Normal distribution of the data was confirmed using the Shapiro-Wilk test. A one-way ANOVA with Dunnett’s multiple comparisons test as a post-test was used to statistically calculate the significant differences for the CFU assay, ELISA, and Luminex. Data are presented as the mean ± standard error of the mean (SEM); *p* < 0.05 is considered to be statistically significant (* *p* < 0.05, ** *p* < 0.01, *** *p* < 0.001, **** *p* < 0.0001). All the data analysis and presentation were done using the Graph Pad Prism 7.0 statistical software (La Jolla, CA, USA).

## Results

### Isolation and Characterization of h-prASC and r-prASC

**Fig. 1:**
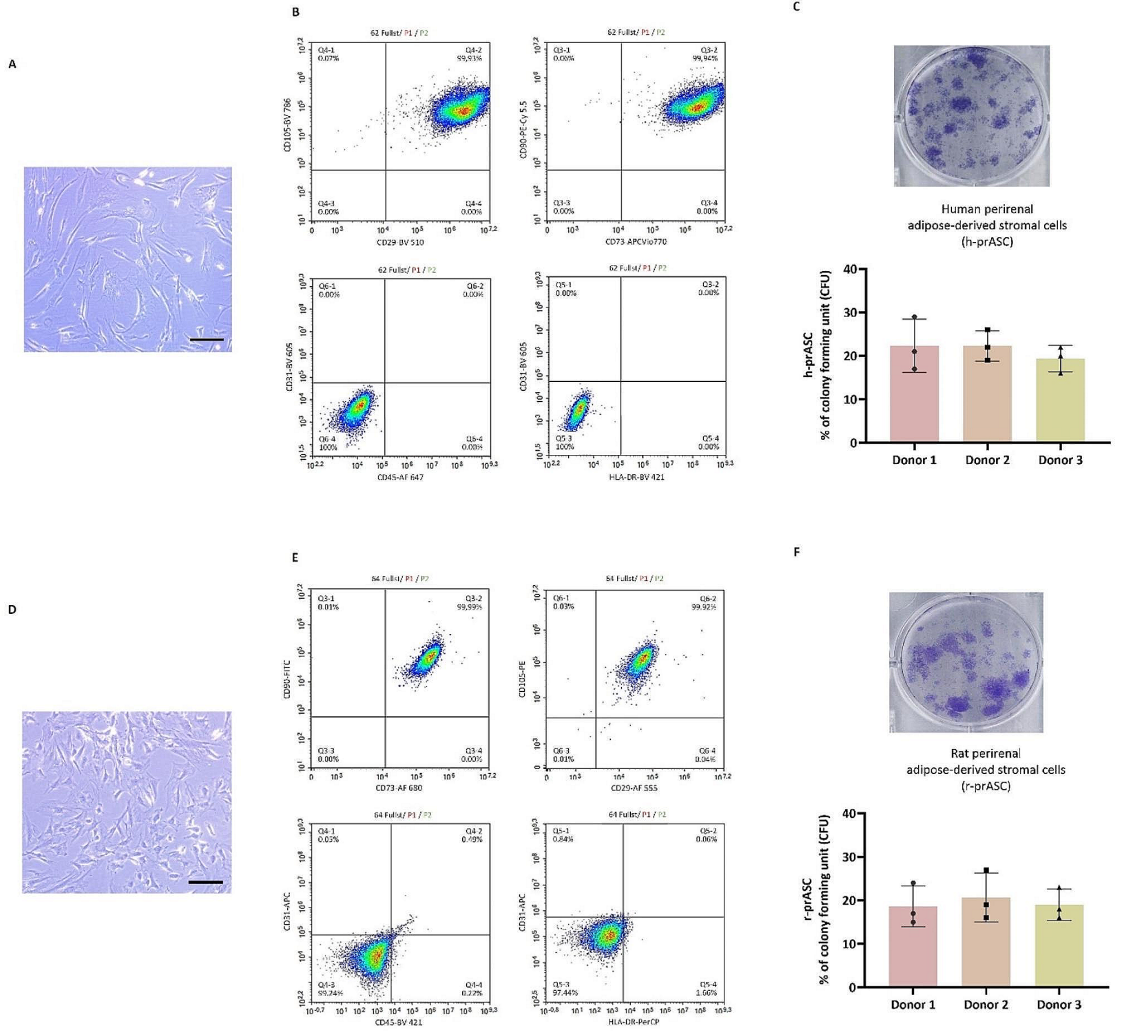
Characterization of human (h-prASC) and rat (r-prASC) perirenal adipose-derived stem cells in vitro. (**A**) Cell morphology of cultured h-prASC (p3) (Objective 20×, scale bar = 20 μm); (**B**) Cell morphology of cultured r-prASC (p3) (bar: 100 μm); (**C**) Representative flow cytometric histograms of CD29, CD90, CD105, CD44, CD45, CD31, CD146, CD11b expression of h-prASC ; (**D**) Representative flow cytometric histograms of CD29, CD90, CD45, MHC Class II, CD106 expression of r-prASC ; (**E**) colony forming unit assay (CFU) of h-prASC ; (**F**) colony forming unit assay (CFU) of r-prASC. For the CFU data, data represent mean values ± SD of 3 individual donors tested in triplicates

For the isolation of h-prASC, an average of 60-gram perirenal adipose tissue was used, yielding around 5.5 × 10^8^ cells ± 2.6 × 10^8^ cells (passage 0). Approximately 70% of the isolated cells did not adhere to the cell culture flask and were aspirated with the first washing after 24 h of culture. Adherent h-prASC cultured in a 25 cm^2^ cell culture flask (T25) reached subconfluence (80%) in 5 to 7 days. These cells showed the typical spindle-shaped morphology (Fig. 1A). The attached cells formed a monolayer composed of cells with a homogeneous morphology.

For the isolation of r-prASC, an average of 12-gram perirenal adipose tissue was used, which resulted in an average yield of 2.1 × 10^8^ ± 0.6 × 10^8^ cells. Similar to the human cells, 60% of the isolated cells did not adhere to the culture flask and were washed away after 24 h. Adherent r-prASC reached confluence (80%) in 3 to 5 days. They displayed similar spindle-shaped morphology and showed to be homogeneous when growing in a monolayer (Fig. 1B). Once confluency was reached, human and r-prASC were subcultured (passage 1). These were cultured until confluence and divided into two T25 flasks also until confluence. At this time, an average of 3.4 × 10^5^ cells were seeded from one T25 into a 75 cm^2^ cell culture flask (T75) (4500 cells/cm^2^ growth area). After reaching 80% confluence, the cells that grew in the T75 flask (passage 3) were used for characterization.

Flow cytometric analysis was performed to determine the cells’ immunophenotype and to eliminate the potential presence of other cell types in the culture. Cultured h-prASC were positive for the mesenchymal stromal cell (MSC) markers CD29, CD90, CD73, and CD105 and did not express the leukocyte and endothelial cell-related markers CD31, CD45, HLA-DR, and CD11b (representative flow cytometry graphs are shown in Fig. 1C). Cultured r-prASC were positive for the same markers (CD29, CD90, CD73, and CD105) and negative also for the same markers (CD31, CD45, and HLA-DR, CD11b) (representative flow cytometry graphs are shown in Fig. 1D). The data showed that both h-prASC and r-prASC cultures possessed the immunophenotype suggested for multipotent stromal cells and were not contaminated with other cell types.

ASC can generate colonies when plated at a low density, i.e., colony forming unit (CFU) capacity. Such proliferative capacity can be assessed by the CFU assay, which provides a quantitative measure of stemness within a given cell population. The number of colonies formed by h-prASC (*n* = 3) and r-prASC (*n* = 3) was comparable, meaning that in human and rat prASC, the stemness capacity was similar and therefore does not seem to be species-dependent (Fig. 1E-F).

**Fig. 2:**
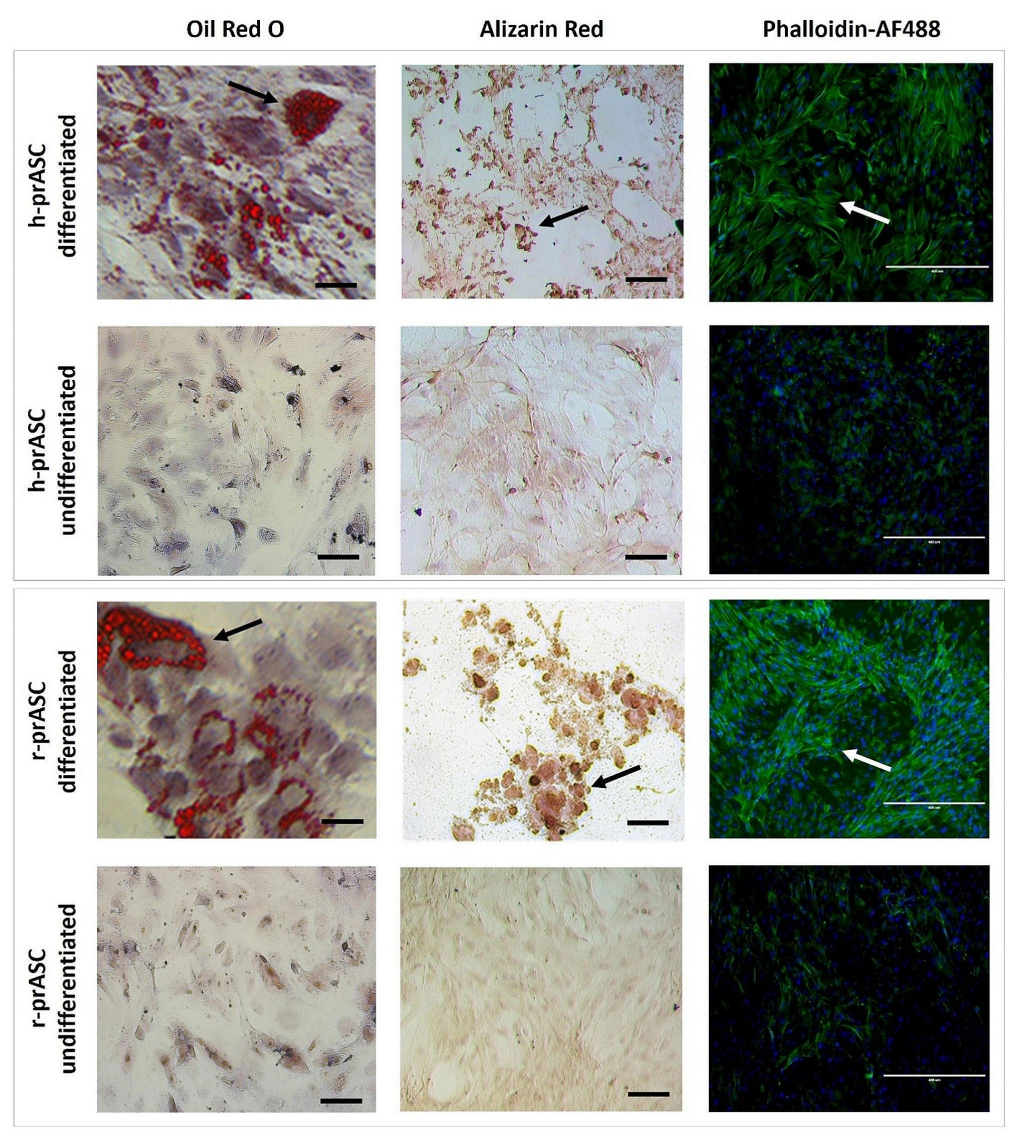
Trilineage differentiation of cultured human (h-prASC) and rat (r-prASC) adipose-derived stem cells. Differentiation into adipocytes, osteoblasts, and smooth muscle cells was induced by adipogenic, osteogenic, and myogenic differentiation medium for 14 days. Undifferentiated cells were cultured in a standard medium (STD-M (+)) for 14 days. After 14 days of incubation in either STD-M (+) or differentiation medium, cultures were stained with Oil Red O (scale bar: 100 μm), Alizarin Red (scale bar: 100 μm), or Phalloidin-AF488 in DAPI (scale bar: 400 μm)

The cells’ differentiation capacity was also investigated. Human and rat prASC (*n* = 3) were capable of adipogenic, osteogenic, and myogenic differentiation. Figure 2 illustrates the differentiation potential from 1 donor; the other donors showed similar results. Upon culture in adipogenic medium, Oil Red-O-stained human and rat prASC revealed multiple intracellular lipid droplets accumulated in intracellular vacuoles. Upon culture in osteogenic medium, Alizarin-Red staining showed Ca^2+^ deposits, indicating differentiation into osteoblast-like cells. Upon culture in a myogenic medium, cells stained with phalloidin demonstrated high F-actin assembly consistent with the characteristics of smooth muscle cells.

### Human and Rat prASC Secretome Profiling Using Mass Spectrometry

Mass spectrometry was performed to describe the secretion profile of human and rat-derived prASC. Secretomes obtained from human and rat prASC exposed to various culturing strategies for 72 h were investigated.

#### Human and Rat prASC Secretomes’ Profiles are Influenced by Different Culturing Strategies

**Fig. 3:**
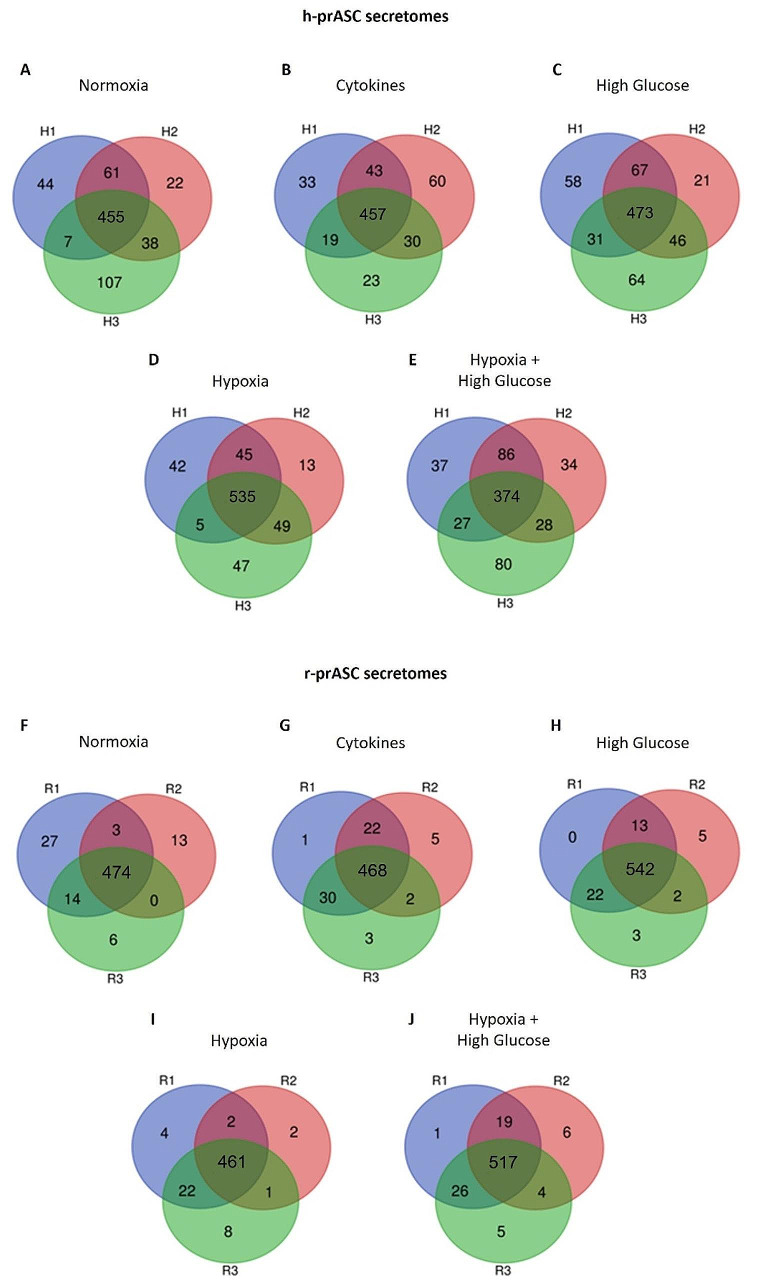
Venn diagrams illustrate the overlap between human (h-prASC) and rat (r-prASC) adipose-derived stem cells’ secretomes from different donors upon different culturing strategies. h-prASC secretomes upon normoxia (**A**), cytokines (**B**), high glucose (**C**), hypoxia (**D**), and hypoxia + high glucose (**E**). r-prASC secretomes upon normoxia (**F**), cytokines (**G**), high glucose (**H**), hypoxia (**I**), and hypoxia + high glucose (**J**)

In the h-prASC secretomes resulting from normoxia, cytokine exposure, high glucose, hypoxia, and hypoxia combined with high glucose, 686, 667, 701, 874, and 611 secretory proteins were identified, respectively (for protein catalog, see Supplementary File 1: S1 – S5). r-prASC secretomes contained 557, 536, 603, 519, and 583 secretory proteins, respectively (for the protein catalog, see Supplementary File 2: S1–S5). We found that 455 (66.32%) (normoxia), 457 (68.51%) (cytokine exposure), 473 (67.47%) (high glucose), 535 (61.21%) (hypoxia), and 374 (61.21%) (hypoxia with high glucose) secretory proteins were common to the three h-prASC cultures of each condition (for the protein catalog, see Supplementary File 1: S6–S10; quantitative overview in Table 1 and representative diagrams in Fig. 3A-E). In the r-prASC cultures, 474 (85.09%) (normoxia), 468 (87.31%) (cytokine exposure), 542 (89.88%) (high glucose), 461 (88.82%) (hypoxia), and 517 (88.67%) (hypoxia with high glucose) secretory proteins were common to all donors of each condition (for the protein catalog, see Supplementary File 2: S6–S10; quantitative overview in Table 2 and representative diagrams in Fig. 3F-J).

**Table 1:** h-prASC secretomes quantitative overview

	Normoxia	Cytokines	High Glucose	Hypoxia	Hypoxia + High Glucose
Total proteins identified per condition	686	667	701	874	611
Proteins common to all samples (% from total proteins identified/condition)	455 (66.32%)	457 (68.51%)	473 (67.47%)	535 (61.21%)	374 (61.21%)
Differentially secreted proteins (% from total proteins identified/condition)		164 (35.88%)	95 (20.08%)	148 (27.66%)	55 (14.70%)
Basal secretion (% from the sum of all proteins identified)	185 (8.06%)				

“Total proteins identified / condition” is the sum of the total number of proteins identified in all three donors analyzed. “Proteins common to all samples” is the total number of proteins present in all three donors. “Differentially secreted proteins” is the number of unique proteins found in a secretome compared to the standard (normoxic) secretome. “Basal secretion” corresponds to the number of proteins found in all secretomes regardless of the culturing condition employed

**Table 2:** r-prASC secretomes quantitative overview

	Normoxia	Cytokines	High Glucose	Hypoxia	Hypoxia + High Glucose
Total proteins identified per condition	557	536	603	519	583
Proteins common to all samples (% from total proteins identified/condition)	474 (85.82%)	468 (87.31%)	542 (89.88%)	461 (88.82%)	517 (88.67%)
Differentially secreted proteins (% from total proteins identified/condition)		50 (10.6%)	104 (26.4%)	163 (37.1%)	165 (35%)
Basal secretion (% from the sum of all proteins identified)	384 (15.59%)				

“Total proteins identified / condition” is the sum of the total number of proteins identified in all three donors analyzed. “Proteins common to all samples” is the total number of proteins present in all three donors. “Differentially secreted proteins” is the number of unique proteins found in a secretome compared to the standard (normoxic) secretome. “Basal secretion” corresponds to the number of proteins found in all secretomes regardless of the culturing condition employed

**Fig. 4:**
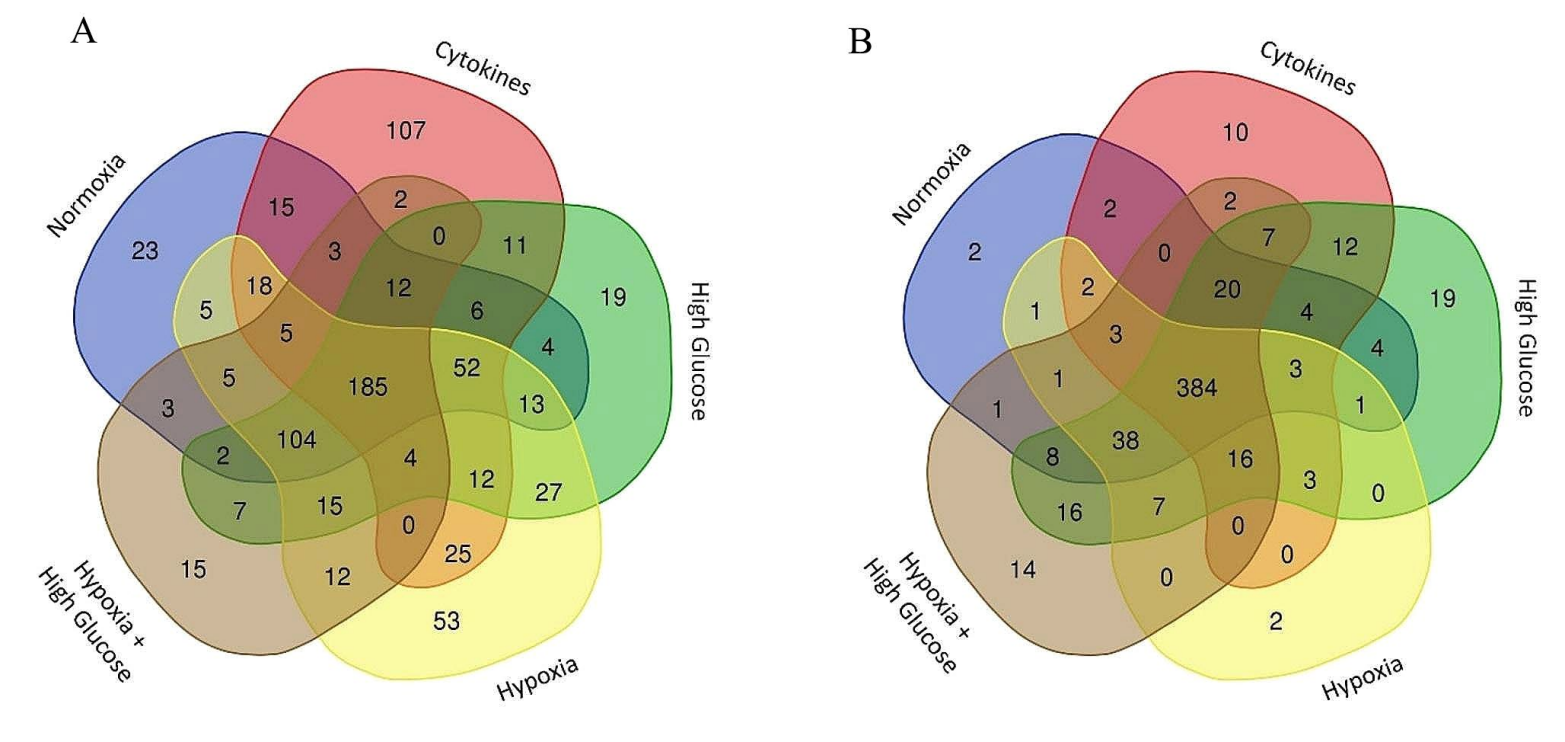
Veen diagrams showing overlaps between h-prASC and r-prASC secretomes from all investigated culturing conditions. h-prASC secretomes (**A**), and r-prASC secretomes (**B**) overlap. N, C, HG, H, and HHG represent the secretomes resulting from the different prASC culturing conditions: normoxia (N), cytokines (**C**), high glucose (HG), hypoxia (H), and hypoxia + high glucose (HHG).

Of particular interest are the proteins present in the h-prASC secretomes regardless of the culturing strategy (Fig. 4). We identified 185 common proteins in all h-prASC secretomes, corresponding to 8.06% of the total proteins described across all conditions (Fig. 4A; Supplementary File 1: S11). The r-prASC secretome, in turn, consisted of 384 common proteins across all culturing conditions investigated, representing 15.59% of the total identified proteins (Fig. 4B; Supplementary File 2: S11).

#### Normoxic Culturing Resulted in Human and Rat prASC Secretomes with Similar Functional Characteristics

Functional annotation clustering using the gene annotation and analysis resource Metascape was performed for human and rat prASC normoxic secretomes to gain insight into the pathway enrichment of these secretomes under standard culturing conditions.

The analysis of the h-prASC normoxic secretome using Metascape revealed various pathway enrichment clusters (for the top 5 most enriched pathways, see Table 3; for the top 100, see Fig. S1). The most enriched clusters included pathways associated with the ECM organization and composition (e.g., NABA CORE MATRISOME, supramolecular fiber organization, collagen formation, regulation of extracellular matrix disassembly, and others), platelet degranulation, regulation of insulin-like growth factor (IGF) transport and uptake by insulin-like growth factor binding proteins (IGFBP), and neutrophil degranulation. Other pathways of interest that were recurrent within the h-prASC normoxic secretome are the ones involved with the immune system (e.g., complement and coagulation cascades, signaling by interleukins, regulation of defense response, and others) and with vascular development (e.g., VEGFA-VEGFR2 signaling pathway, regulation of angiogenesis, and blood vessel development). Proteins overrepresented by these clusters include various collagens (COL1A1, COL1A2, COL3A1, COL4A1, COL4A2, and others), fibronectin, laminins (laminin-1 subunit beta, laminin-1 subunit gamma, laminin-14 subunit alpha, and others), IGFBP (IGFBP3, 5, and 7), ILs (IL-1 receptor 3 and IL-6), complement components (complement C3, C4-A, C1r, C1s, C5, and others), vascular peroxidase 1, pantetheine hydrolase, pantetheinase, spondin-1, and serpins (serpin C1, A3, F2, and others) (for the h-prASC normoxia-dependent secretory protein catalog see Supplementary File 1: S6).

**Table 3:** The top 5 pathway and process enrichment clusters of human and rat pr-ASC (h-prASC and r-prASC) normoxic secretome using Metascape

	Process Enrichment Clusters	GO	Count	%	-log10(*P*)
h-prASC Normoxic secretome	NABA CORE MATRISOME	M5884	75	16.59	72.00
	Platelet degranulation	R-HSA-114,608	53	11.73	61.70
	Regulation of Insulin-like Growth Factor (IGF) transport and uptake by Insulin-like Growth Factor Binding Proteins (IGFBPs)	R-HSA-381,426	44	9.73	47.59
	Neutrophil degranulation	R-HSA-6,798,695	70	15.49	46.96
	Supramolecular fiber organization	GO:0097435	70	15.49	42.60
r-prASC Normoxic secretome	Innate immune system	R-RNO-168,249	104	24.53	51.53
	Extracellular matrix organization	R-RNO-1,474,244	42	9.91	28.71
	supramolecular fiber organization	GO:0097435	66	15.57	28.18
	Platelet degranulation	R-RNO-114,608	30	7.08	23.80
	Regulation of Insulin-like Growth Factor (IGF) transport and uptake by Insulin-like Growth Factor Binding Proteins (IGFBPs)	R-RNO-381,426	28	6.60	22.77

“GO” is the universal identifier of the described ontology term. “Count” is the number of proteins described in the respective secretome present in the given ontology term. “%” is the percentage of all of the input proteins that are found in the given ontology term. “-log10(P)” is the negative p-value (vs. counts expected by chance) in log base 10

The r-prASC normoxic secretome analysis also revealed various pathway enrichment clusters (for the top 5 most enriched pathways, see Table 3; for the top 100, see Fig. S6). Similar to the h-prASC normoxic secretome, the r-prASC normoxic secretome displayed a large number of ECM-related pathways (extracellular matrix organization, and supramolecular fiber organization, represented by proteins such as fibronectin, and collagens (COL1A1, COL1A2, COL1A3, COL2A1, and others). The pathways of platelet degranulation and regulation of IGF transport and uptake by IGFBP were also highly enriched, as in the h-prASC normoxic secretome. The innate immune system was also highly enriched in the r-prASC normoxic secretome, represented by proteins such as IL1R1, IL6, and complement components (C1s, C1r, C3, C4, and others). The r-prASC normoxic secretome was also enriched with proteins in the pathways of protein folding (represented by various heat shock proteins (HSP), SPARC, clusterin, and others) and proteins in the pathway response to growth factors (LTBP-1 and 2, 14-3-3 protein beta/alpha, PDGFR-like protein, VEGF-D, and others). For the r-prASC normoxia-dependent secretory protein catalog, see Supplementary File 2: S6. These data demonstrate that human and rat prASC secretomes resulting from normoxic culturing share many functional similarities.

### Alternative Culturing Strategies Affect the prASC Secretome

Various culturing strategies have been used to generate diverse secretomes. Human and rat prASC were exposed to cytokines, high glucose concentrations, hypoxia, and a combination of hypoxia and high glucose for 72 h. Subsequently, we investigated the presence of differentially secreted proteins by comparing them to their respective prASC normoxic secretome. Finally, we analyzed the pathways to which these differentially secreted proteins belonged per strategy.

#### Different Culturing Strategies Affect the h-prASC Secretomes’ Protein and Functional Profiles

##### Cytokines and High Glucose-dependent h-prASC Secretomes

The different culturing strategies had an impact on the total number of secretory proteins observed. Each condition exhibited secretomes with distinct proteins compared to the normoxia-dependent secretome. Exposure to cytokines and high glucose altered the composition of h-prASC secretome compared to standard normoxic culturing, and 164 and 95 differentially secreted proteins were identified, respectively, accounting for 35.88% and 20.08% of the total proteins identified per condition (Table 1). The cytokines-dependent h-prASC secretome was composed of pathways associated with the metabolism of amino acids (including the pathway of asparagine N-linked glycosylation), vascular development (VEGFA-VEGFR2 signaling, VEGFR1 2, and blood vessel endothelial cell migration), carbohydrate metabolic process, and protein processing in the endoplasmic reticulum (for the top 5 most enriched pathways, see Table 4; for the top 100, see Fig. S2). Immune system-related pathways were also consistently displayed as enriched in this secretome (e.g., neutrophil degranulation, MHC class II antigen presentation, IL17 signaling, regulation of immune effector process, and others). Cytokine-dependent secretory proteins included proteasome subunits, CD166 antigen, C-X-C motif chemokine 1 and 5, annexins 3, 7, and 11, and the adhesion molecule Von Willebrand factor A domain-containing protein 5 A (for the h-prASC cytokine-dependent secretory protein catalog see Supplementary File 1: S7).

**Table 4:** Top 5 pathway and process enrichment clusters of h-prASC differentially secreted proteins per condition using Metascape

	Process Enrichment clusters	GO	Count	%	-log10(*P*)
Cytokines (h-prASC)	Metabolism of amino acids	R-HSA-71,291	25	15.34	19.37
	VEGFA-VEGFR2 signaling pathway	WP3888	18	11.04	10.58
	Carbohydrate metabolic process	GO:0005975	18	11.04	10.03
	Protein processing in endoplasmic reticulum	hsa04141	12	7.36	9.77
	Asparagine N-linked glycosylation	R-HSA-446,203	14	8.59	8.89
High Glucose (h-prASC)	Axon guidance	R-HSA-422,475	21	22.11	16.40
	Neutrophil degranulation	R-HSA-6,798,695	18	18.95	13.92
	Amino acid metabolism	WP3925	8	8.42	9.31
	Metabolism of carbohydrates	R-HSA-71,387	11	11.58	8.61
	Parkin-ubiquitin proteasomal system pathway	WP2359	7	7.37	8.56
Hypoxia (h-prASC)	Neutrophil degranulation	R-HSA-6,798,695	30	20.27	23.73
	Axon guidance	R-HSA-422,475	26	17.57	17.51
	Asparagine N-linked glycosylation	R-HSA-446,203	16	10.81	11.60
	Metabolism of carbohydrates	R-HSA-71,387	14	9.46	9.61
	Parkin-ubiquitin proteasomal system pathway	WP2359	8	5.41	8.65
Hypoxia + High Glucose (h-prASC)	Neutrophil degranulation	R-HSA-6,798,695	16	29.09	15.63
	Regulation of Insulin-like Growth Factor (IGF) transport and uptake by Insulin-like Growth Factor Binding Proteins (IGFBPs)	R-HSA-381,426	6	10.91	6.96
	Formation of the cornified envelope	R-HSA-6,809,371	6	10.91	6.86
	Cell-cell adhesion	GO:0098609	9	16.36	6.33
	NABA MATRISOME ASSOCIATED	M5885	10	18.18	6.04

“GO” is the universal identifier of the described ontology term. “Count” is the number of proteins described in the respective secretome present in the given ontology term. “%” is the percentage of all of the input proteins that are found in the given ontology term. “-log10(P)” is the negative p-value (vs. counts expected by chance) in log base 10

The high glucose-dependent secretome displayed pathways of axon guidance, neutrophil degranulation, protein and carbohydrate metabolism, and the ubiquitin-proteasome system (for the top 5 most enriched pathways, see Table 4; for the top 100, see Fig. S3). Proteins included in pathways of axon guidance are, for instance, CXCL12, and GDF7, while proteins included in the neutrophil degranulation pathway are, for instance, various histones, MMP-2 and − 9, and proteins included in the pathways of amino acid and carbohydrate metabolism are many metabolism-related enzymes (hexokinase, pyruvate kinase, glucose-6-phosphatase, deaminases, decarboxylases, oxidases. Ubiquitins, PAI-2, HSP, and proteasome regulatory particles are included in the Parkin-ubiquitin proteasomal system pathway. Proteins, such as the glutamine synthetase, annexin A4, and TGF-β, present in are involved in the regulation of IGF transport and uptake by IGFBP. This pathway was further enriched when compared to the h-prASC normoxic secretome. Moreover, compared to the normoxic secretome, the h-prASC high glucose-dependent secretome displayed, although to a much lesser extent, enrichment of pathways of the immune system (such as signaling by interleukins), extracellular matrix (such as extracellular matrix organization), and vascular development (such as VEGFA-VEGFR2 signaling pathway) (for the h-prASC high glucose-dependent secretory protein catalog, see Supplementary File 1: S8).

#### Hypoxia and Hypoxia + High Glucose-Dependent h-prASC Secretomes

When compared to the standard culturing under normoxic conditions, h-prASC secretomes resulting from hypoxia and hypoxia + high glucose exposure displayed 148 and 55 differentially secreted proteins, which corresponds to 27.66% and 14.70% of the total number of identified proteins per condition, respectively (Table 1).

Hypoxia-dependent proteins displayed enrichment in clusters of neutrophil degranulation, axon guidance, asparagine N-linked glycosylation, metabolism of carbohydrates, and the ubiquitin-proteasome system (for the top 5 most enriched pathways, see Table 4; for the top 100, see Fig. S4). Identified proteins included IGFBP3, IGFBP4, MMP-2 and − 9, collagens type IV alpha 1 and 5, and annexin A1 – which clustered within the pathways of neutrophil degranulation and axon guidance –, various molecular chaperones (HSPA8, HSP90, HSP70, and HSP27), the protein disulfide-isomerase, and proteasome subunits – as part of the Parkin-ubiquitin proteasomal system pathway – and metabolic enzymes (hexokinase, pyruvate kinase, glucose-6-phosphatase) that were clustered in the pathway of metabolism of carbohydrates. Moreover, vascular development-related proteins (associated with the VEGFA-VEGFR2 signaling pathway), immune system-related proteins (IL and complement proteins), and ECM proteins were also detected (for the h-prASC hypoxia-dependent secretory protein catalog, see Supplementary File 1: S9).

ASC cultured under exposure to a combination of hypoxia and high glucose resulted in the enrichment of neutrophil degranulation, regulation of IGF transport and uptake by IGFFBPS, formation of the cornified envelope, cell-cell adhesion and NABA MATRISOME ASSOCIATED pathways (for the top 5 most enriched pathways, see Table 4; for the top 100, see Fig. S5). Secretory proteins that were identified include various collagen types (type I alpha 1 and 2, type III alpha 1, type V alpha 2 and 1, and others), adhesion molecules (integrins, selectins, and proteoglycans), VEGFA, and galactosidase beta 1 (for the h-prASC hypoxia + high glucose-dependent secretory protein catalog see Supplementary File 1: S10). Pathways of transforming growth factor beta receptor signaling and positive regulation of growth were also enriched in this secretome.

#### Different Culturing Strategies Affect the r-prASC Secretomes’ Protein and Functional Profiles

##### Cytokines and High Glucose-Dependent r-prASC Secretomes

Different culturing strategies have unique effects on r-prASC secretomes’ protein and functional profiles. Cytokines and high glucose exposure also significantly affected the r-prASC secretomes compared to normoxic conditions. Specifically, cytokines led to 50 (10.6%) differentially secreted proteins, while high glucose exposure resulted in 104 (26.4%) differentially secreted proteins (Table 2). The functional analysis revealed that proteins uniquely identified in the r-prASC cytokines-dependent secretome mostly clustered in pathways associated with the tryptophan metabolism, response to xenobiotic stimulus, matrix metalloproteinases, proteasome degradation, and positive regulation of proteins localization to the nucleus (for the top 5 most enriched pathways, see Table 5; for the top 100, see Fig. S7). Proteins in this secretome included the tryptophan and aspartate-tRNA ligase, class I histocompatibility antigen Non-RT1.A alpha-1 chain, TNF-α, IFN-γ, MMP-11 and 13, and some proteasome subunits (for the r-prASC cytokines-dependent secretory protein catalog, see Supplementary File 2: S7).

**Table 5:** Top 5 pathway and process enrichment of r-prASC differentially secreted proteins per condition using Metascape

	Process Enrichment Cluster	GO	Count	%	-log10(*P*)
Cytokines (r-prASC)	Tryptophan metabolism	WP270	5	11.36	7.37
	Response to xenobiotic stimulus	GO:0009410	11	25.00	6.24
	Matrix metalloproteinases	WP278	3	6.82	5.00
	Proteasome degradation	WP302	3	6.82	3.96
	Positive regulation of protein localization to nucleus	GO:1,900,182	4	9.09	3.93
High Glucose (r-prASC)	Tryptophan metabolism	WP270	5	7.25	6.37
	Regulation of Insulin-like Growth Factor (IGF) transport and uptake by Insulin-like Growth Factor Binding Proteins (IGFBPs)	R-RNO-381,426	6	8.70	5.81
	Response to xenobiotic stimulus	GO:0009410	13	18.84	5.74
	positive regulation of establishment of protein localization	GO:1,904,951	10	14.49	5.74
	Response to estradiol	GO:	8	11.59	5.27
	Proteasome degradation	WP302	4	5.80	4.87
Hypoxia (r-prASC)	Neutrophil degranulation	R-RNO-6,798,695	13	18.84	6.12
	Cellular response to stress	R-RNO-2,262,752	10	14.49	5.86
	Platelet degranulation	R-RNO-114,608	10	14.49	5.86
	Supramolecular fiber organization	GO:0097435	6	8.70	5.89
	Gluconeogenesis	R-RNO-70,263	4	9.09	3.96
Hypoxia + High Glucose (r-prASC)	Innate immune system	R-RNO-168,249	19	19.59	7.98
	Regulation of Insulin-like Growth Factor (IGF) transport and uptake by Insulin-like Growth Factor Binding Proteins (IGFBPs)	R-RNO-381,426	8	8.25	7.40
	Cellular response to stress	R-RNO-2,262,752	14	14.43	7.12
	Hemostasis	R-RNO-109,582	13	13.40	6.28
	Protein folding	GO:0006457	9	9.28	6.19

“GO” is the universal identifier of the described ontology term. “Count” is the number of proteins described in the respective secretome present in the given ontology term. “%” is the percentage of all of the input proteins that are found in the given ontology term. “-log10(P)” is the negative p-value (vs. counts expected by chance) in log base 10

The high glucose-dependent proteins were clustered in groups of proteins associated with pathways of tryptophan metabolism, regulation of IGF transport and uptake by IGFBP, response to xenobiotic stimulus, and proteasome degradation (for the top 5 most enriched pathways, see Table 5; for the top 100, see Fig. S8). Proteins such as various collagens, fibulin-1, complement components, metalloproteinase inhibitor 1, histones isoforms, and nucleolin were present (for the r-prASC high glucose-dependent secretory protein catalog, see Supplementary File 2: S8).

#### Hypoxia and Hypoxia + High Glucose-Dependent r-prASC Secretomes

Hypoxia and hypoxia + high glucose culturing resulted in more differentially secreted proteins, 163 and 165, respectively, compared to the cytokine and high glucose secretome. This accounted for 37.1% and 35% of the proteins identified in each secretome (Table 2). Proteins differentially secreted under hypoxia culturing clustered in pathways of neutrophil degranulation, cellular response to stress, platelet degranulation, supramolecular fiber organization, gluconeogenesis, and others (for the top 5 most enriched pathways, see Table 5; for the top 100, see Fig. S9). Proteins included various collagens, moesin, fibulin-1, annexin, b-FGF, HGF, PDGF-C, and C-C motif chemokine 20 (for the r-prASC hypoxia-dependent secretory protein catalog, see Supplementary File 2: S9).

The prASC secretome resulting from hypoxia + high glucose culturing displayed enrichment in pathways of the innate immune system, regulation of IGF transport and uptake by IGFBPs, cellular response to stress, hemostasis, protein folding, and others (for the top 5 most enriched pathways, see Table 5; for the top 100, see Fig. S10). Proteins within these pathways include prohibitin 1, thrombospondin-4, TNF-α, MMP- 9 and 13, IGFBP-6, and others (for the r-prASC hypoxia + high glucose-dependent secretory protein catalog, see Supplementary File 2: S10). We also observed the consistent presence of pathways associated with protein and metabolic regulation in this secretome: protein catabolic process, peptide metabolic process, peptide catabolic process, hormone metabolic process, regulation of protein stability, proteasome degradation, and Golgi organization. Various enzymes (proteases, aminopeptidases) and ubiquitins were identified within these pathways.

### Human and Rat prASC Secretome Profiling Using ELISA and Luminex

To confirm the presence and determine the concentration of key proteins associated with critical biological processes related to ASC, such as angiogenesis, ECM composition, and the immune system, Luminex and ELISA assays were conducted on both human and rat pr-ASC secretomes resulting from various culturing strategies. VEGF, PDGF, and b-FGF were chosen to represent angiogenic processes [12, 18]. Collagen I alpha I represented the most abundant and consistently present ECM component [35]. IL-1α and IL-10 represented both anti- and proinflammatory factors associated with the immune system pathways [36].

**Fig. 5:**
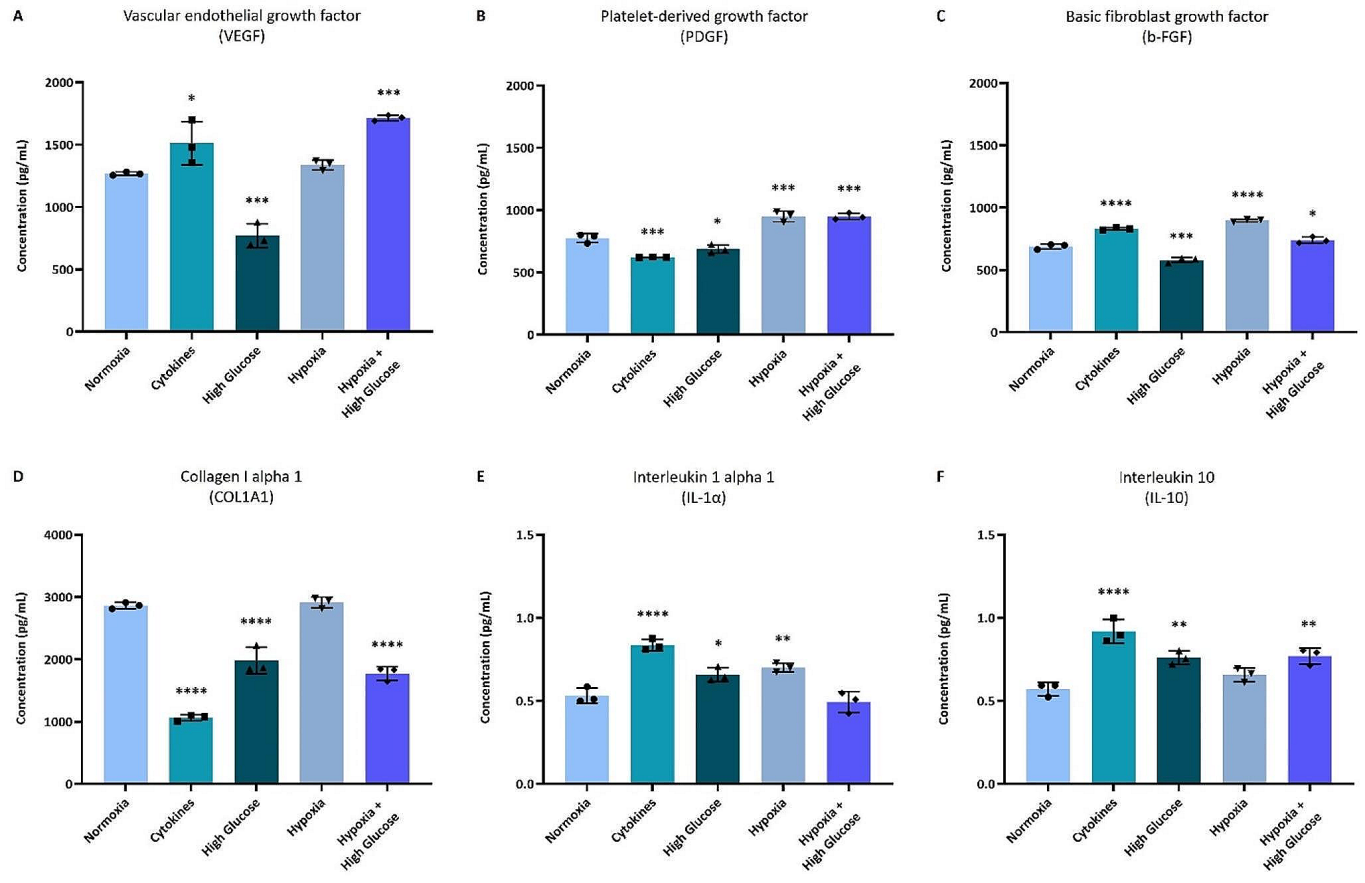
VEGF (**A**), PDGF (**B**), b-FGF (**C**), collagen I alpha I (**D**), IL-1α (**E**), IL-10 (**F**) h-prASC secretion after 72 h exposure to normoxia (21% O_2_), cytokines, high glucose, hypoxia, hypoxia + high glucose. A one-way ANOVA with Dunnett’s multiple comparisons test was performed to analyze significant differences compared to the normoxia group (*). Data represent mean values ± SD of 3 pooled samples measured in triplicate. ****p < 0.0001; *** *p* < 0.001; ** *p* < 0.01, *p < 0.05

All the angiogenic key factors investigated were present in all h-prASC secretomes analyzed (Fig. 5). The highest levels of VEGF were found in the cytokines and hypoxia + high glucose secretomes (*p* < 0.05 and *p* < 0.001) as compared with the normoxic secretome (Fig. 5A). A significant reduction in VEGF levels was observed in the high glucose-derived h-prASC secretome (*p* < 0.001) as compared with the normoxic culturing (Fig. 5A). PDGF levels were increased in the secretomes of hypoxia and hypoxia + high glucose culturing (*p* < 0.001) as compared with the normoxic secretome (Fig. 5B). On the other hand, cytokines and high glucose culturing decreased levels of PDGF (*p* < 0.001 and *p* < 0.05) compared to normoxic culturing (Fig. 5B). h-bFGF concentrations were increased by cytokines (*p* < 0.0001), hypoxia (*p* < 0.0001), and hypoxia + high glucose (*p* < 0.05) culturing, while high glucose reduced bFGF (*p* < 0.001) when compared to normoxic culturing (Fig. 5C). h-prASC normoxic secretomes exhibited high concentrations of collagen I alpha I (Fig. 5D). Cytokines, high glucose, and hypoxia + high glucose culturing significantly reduced the secretion of this protein by h-prASC (*p* < 0.0001) (Fig. 5D). The immune system-related factors IL-1α and IL-10 were detected at low levels in all h-prASC secretomes (Fig. 5E-F). IL1α, a proinflammatory cytokine, was increased by cytokines, high glucose, and hypoxia culturing (*p* < 0.0001, *p* < 0.05, and *p* < 0.0001, respectively) when compared to normoxic culturing (Fig. 5E). IL-10, an anti-inflammatory cytokine, was increased by the cytokines, high glucose, and hypoxia + high glucose culturing (*p* < 0.0001, *p* < 0.01, and *p* < 0.01, respectively) when compared to the normoxic culturing (Fig. 5F).

**Fig. 6:**
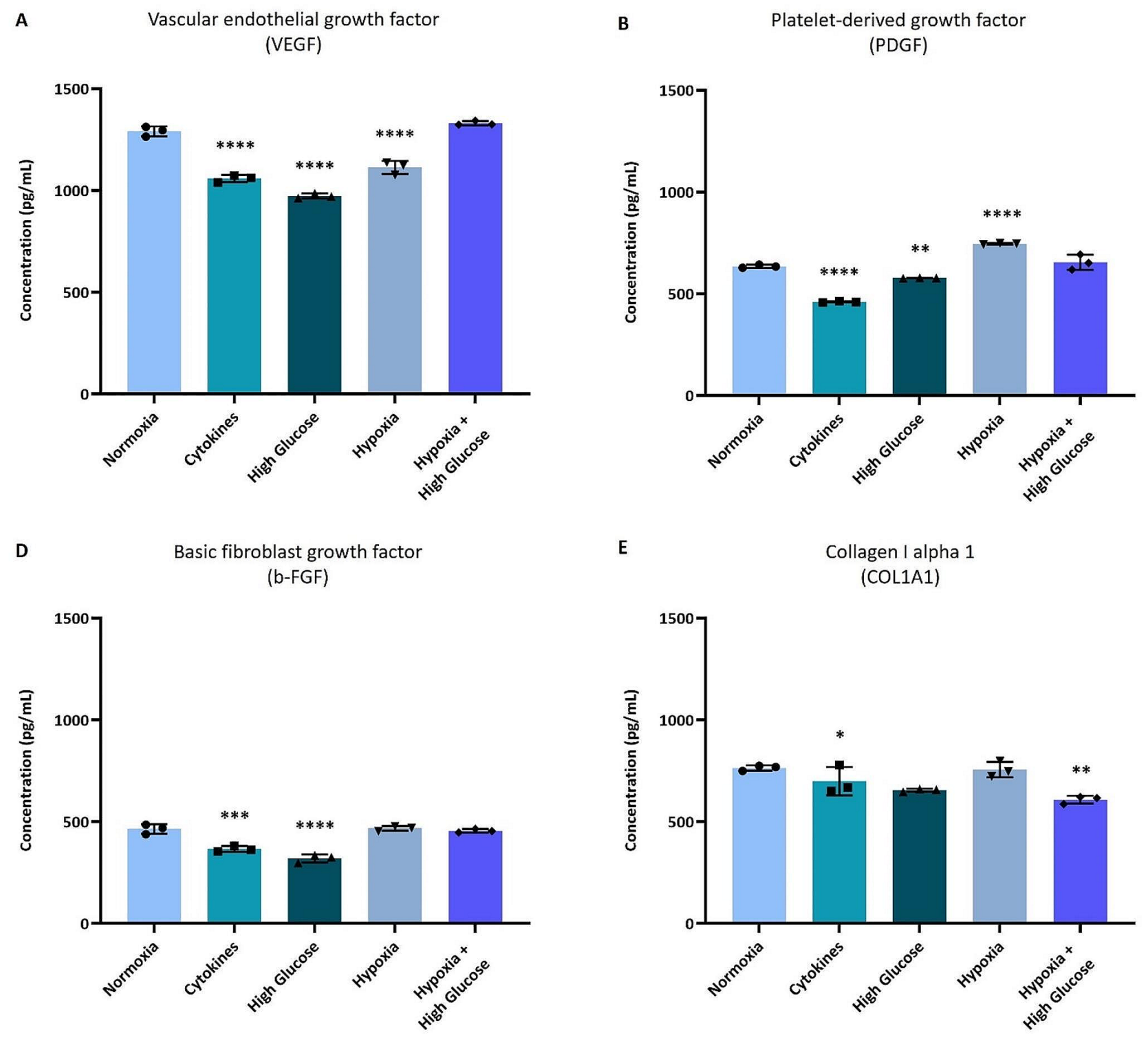
Effects of different culturing conditions on the quantification of paracrine factors in r-prASC secretomes. VEGF (**A**), PDGF (**B**), b-FGF (**C**), and collagen I alpha I (**D**) r-prASC secretion after 72 h exposure to normoxia, cytokines, high glucose, hypoxia, hypoxia + high glucose. A one-way ANOVA with Dunnett’s multiple comparisons test was performed to analyze significant differences compared to the normoxia group (*). Data represent mean values ± SD of 3 pooled samples measured in triplicates. ****p < 0.0001; *** *p* < 0.001; ** *p* < 0.01, *p < 0.05

For r-prASC secretomes, the same key proteins were quantified (Fig. 6). Normoxia induced high VEGF concentrations (Fig. 6A). As compared with the normoxia r-prASC secretome, VEGF levels were significantly decreased when the cells were exposed to cytokines (*p* < 0.0001), high glucose (*p* < 0.0001), and hypoxia (*p* < 0.0001) (Fig. 6A). PDGF concentration was increased in the r-prASC secretomes secreted after hypoxia exposure (*p* < 0.0001) and decreased by cytokines (*p* < 0.0001) and high glucose (*p* < 0.01) exposure when compared to normoxia exposure (Fig. 6B). r-bFGF concentrations were significantly decreased by cytokines and high glucose exposure (*p* < 0.001 and *p* < 0.0001, respectively) (Fig. 6C). A reduction in collagen I alpha I was observed in the secretomes of r-prASC exposed to high glucose (*p* < 0.05) and hypoxia + high glucose (*p* < 0.01) (Fig. 6D) when compared to the normoxic secretome. The cytokine IL-1α and IL-10 were not detected in any of the r-prASC secretomes.

## Discussion

The ASC secretome could constitute a promising therapy for a variety of medical conditions. Secretome-based therapies are more attractive than cell-based strategies because they involve fewer ethical concerns, greater production control, ease of storage, and allow modulation by simply modifying cell culturing conditions in vitro. In this study, we characterized human and rat pr-ASC, described their secretomes resulting from the exposure to various culturing conditions – normoxia, cytokines, high glucose, hypoxia, and hypoxia + high glucose –, and confirmed the presence of key proteins essential for regenerative processes. In our study, human and rat pr-ASC that were isolated, cultured, and expanded expressed the expected subset of MSC markers and lacked expression of leukocyte and endothelial cell markers. Moreover, they showed to have stemness and a multipotency potential. The exposure of these cells to different culturing conditions resulted in different protein secretion profiles. Using GO term analysis, we found enrichment of different pathways in the secretomes after different culturing conditions. Our data expand known protein and pathway catalogs by describing culturing-dependent ASC secretion products and their functional profile. We showed that human and rat prASC secrete many beneficial proteins with potential clinical applications depending on the condition employed to culture these cells. Moreover, by revealing functional similarities among rat and human prASC secretomes, we show that pre-clinical results can be extrapolated to future clinical investigations.

Our standard culturing condition (normoxia) displayed, for both species, enrichment in pathways associated with the ECM (such as NABA CORE matrisome, supramolecular fiber organization, and ECM organization), regulation of IGF transport and uptake by IGFBP, and platelet degranulation. ECM-associated proteins have been reported before in various studies investigating the paracrine benefits of ASC from other sources (e.g., subcutaneous and gonadal adipose tissue) [17, 26, 37]. Therefore, this ASC secretome seems to stimulate ECM synthesis and repair. This secretome may thus be a promising strategy in wound healing [13, 27, 38]. The enrichment in proteins involved in the pathway of regulation of IGF transport and uptake by IGFBP suggests that the normoxic secretomes can modulate the IGF system, which plays a central role in ensuring proper cell growth and regulating metabolic homeostasis [39]. IGFs, IGFBPs, and related proteins also modulate angiogenesis [40, 41]. Since other angiogenesis-related pathways were also enriched in the normoxic secretomes, such as the VEGFA-VEGFR2, vascular development, and blood vessel development pathways, this secretome also has a proangiogenic profile. We confirmed the presence of VEGF at significant levels in our secretome. The enrichment of the pathway of platelet degranulation is in line with the presence of significant amounts of PDGF and bFGF in our normoxic secretomes. Also, these proteins are involved in angiogenesis [12, 18, 19].

We aimed to evaluate whether different culturing conditions would result in different secretomes, which could have different effects on regenerative processes and be used for different therapeutic purposes. Our cocktail of cytokines was used to reproduce a proinflammatory milieu that could potentially stimulate a prASC anti-inflammatory secretome. Although not in the top 5 pathways, the human cytokine secretome displayed enrichment in pathways associated with the immune system, such as neutrophil degranulation, MHC class II antigen presentation, or IL-17 signaling. We also found an increase in IL-10 and IL-1α in the human cytokine secretomes compared to the standard normoxic secretome. In the rat cytokine secretome, pathways that indirectly modulate the immune system were enriched, including tryptophan metabolism, proteasome degradation, and response to xenobiotic stimulus. The first and last pathways play a key role in regulating the activation and differentiation of T-cells and in modulating immune tolerance [42, 43], while the “proteasome degradation” pathway regulates the structure and function of key immune system-related proteins [44]. Cytokine exposure has also been associated with the secretion of proangiogenic factors by ASC [25, 45]. This is in line with the enrichment of the “VEGFA-VEGFR signaling, endothelial cell differentiation, and blood vessel endothelial cell migration pathways, as well as the increased VEGF and bFGF concentration in h-prASC cytokines-dependent secretomes versus normoxic secretomes.

We also used high glucose culturing as a stressor of cells. Our concentration of choice (35 mM glucose) reflected a high-end serum glucose level (> 540 mg/dL). Our h-prASC high glucose-dependent secretome displayed enrichment in pathways associated with the immune system (e.g., neutrophil degranulation and signaling by interleukins) and the ECM (e.g., extracellular matrix organization, regulation of supramolecular fiber organization, and response to wounding). Metabolic pathways and their associated proteins were also enriched (pathways of amino acid metabolism, metabolism of carbohydrates, and others). Such pathways are thought to be a reflex of the prASC metabolic plasticity, a capacity that may allow them to match different energy offers and demands [46]. Interestingly, in the h-prASC high glucose secretome, the pathways of axon guidance and Parkin-ubiquitin proteasomal system were highly enriched. The first suggests that this secretome could be involved in neuronal circuit formation [47], and the second is a reflex of the presence of ubiquitin, HSP, and regulatory particles mainly involved in various cellular processes, such as cell cycling progression and various signaling pathways [44, 48]. The r-prASC also produced a high glucose-dependent secretome with an immunomodulatory profile (represented by the pathways of tryptophan metabolism, response to xenobiotic stimulus, proteasome degradation, innate immune system, and others) and a metabolic profile (represented by pathways of amino acid metabolic process, negative regulation of catabolic process and metabolism of steroids). Investigations describing the effects of different (high) glucose concentrations on MSC have been reported with negative or no impact on proliferation rates, differentiation potential, and immunomodulatory properties [49–51].

ASC were also cultured under hypoxic conditions. This culturing condition has been reported to increase the secretion of angiogenic [12] and anti-apoptotic proteins [52]. Although not in the top 5 pathways enriched, various pathways associated with angiogenesis or apoptosis were indeed enriched in the hypoxic-dependent h-prASC secretomes, such as VEGFA-VEGFR2 signaling pathway, regulation of IGF transport and uptake by IGFBP, regulation of intrinsic apoptotic signaling pathways, cellular homeostasis, and others. The angiogenic potential of this secretome is also in line with the increased levels of PDGF and bFGF in this secretome compared with the normoxic secretome. Human prASC hypoxia-dependent secretomes also showed enrichment of immune-associated pathways, such as neutrophil degranulation and innate immune response, and a potential function in modulating metabolic pathways, as the pathways metabolism of carbohydrates and gluconeogenesis were enriched. Moreover, hypoxia also further enriched pathways associated with ECM components, such as NABA matrisome associated, Extracellular matrix organization, and proteoglycan metabolic process. This enrichment is supported by high collagen I alpha I level in this secretome compared to the normoxic secretome.

The r-prASC hypoxia-dependent secretome showed different enrichment pathways than the h-prASC hypoxia-dependent secretome. It contained a more extensive list of enriched pathways, highlighting many pathways associated with oxidative stress and regulation of apoptosis, including response to reactive oxygen species, response to apoptotic signaling pathway, programmed cell death, binding and uptake of ligands by scavenger receptors, regulation of oxidative stress-induced cell death. Angiogenic pathways were less present in this secretome when compared to its human counterpart, with the positive regulation of vascular permeability pathways present at the bottom of the top 100 pathway enrichment.

The combined effect of high glucose and hypoxia was employed to further exacerbate a potentially beneficial response from the prASC, enhancing the response that we had seen upon high glucose and hypoxia separately. Human and rat secretomes exposed to this condition showed enrichment in pathways associated with ECM, as well as regulation of IGF transport and uptake by IGFBP and the immune system. In human secretomes, the pathways of transforming growth factor beta receptor signaling and positive regulation of growth were enriched, suggesting a role for this secretome in cell growth, differentiation, and apoptosis regulation. In the h-prASC secretome, pathways of angiogenesis regulation were also enriched, which is in line with the increased VEGF, PDGF, and bFGF levels found in this secretome compared with the normoxic secretome. On the other hand, r-prASC exposed to hypoxia + high glucose revealed enrichment in pathways involved in regulating metabolism and protein homeostasis (protein catabolic process, peptide metabolic process, protein folding, proteasome degradation, and others). The enhanced metabolic profile of this secretome may be a consequence of the high glucose presence and the high metabolic plasticity intrinsic to stem cells [50, 53, 54]. On the other hand, enrichment in protein homeostasis pathways could result from the cells attempting to regain balance upon exposure to a potent stressor [55], such as the combination of high glucose and hypoxia.

Our study supports the promising therapeutic potential of prASC secretomes in regenerative medicine. This work serves as a solid foundation for future research in this field. However, this analysis relied on in vitro cultured cells, which may not fully replicate in vivo conditions. Including in vivo models in future studies would complement the understanding. Functional validation is critical to establish the actual therapeutic potential of the identified pathways associated with the secretomes investigated in this study. To further corroborate our data, it would be beneficial to evaluate the functionality of the different types of secretomes in specific in vitro tests, such as assays to assess angiogenic potential, the effects on the production and secretion of ECM components, and the influence of the secretomes on immune cell activity. These tests would provide additional insights into the therapeutic potential and mechanisms of action of the secretomes. Including such functional assays in future studies would help validate and expand upon our findings, highlighting the differential effects and potential advantages of various prASC-derived secretomes. Additionally, this study focused specifically on perirenal ASC, limiting its direct applicability to other ASC types. Exploring the secretome of ASC from different adipose tissue depots would be valuable. Furthermore, there are limitations in protein detection range and potential biases in protein identification when using mass spectrometry-based proteomics. Further refinements and complementary approaches can enhance the understanding of the pathways involved.

On a positive note, we must highlight the advantages of secretomes as a cell-free product. They can be produced from non-autologous perirenal fat-derived stromal cells in bulk and stored for an extended period, ensuring a stable supply. In addition, perirenal AT, which is often discarded after kidney surgeries, offers a cost-effective and ethical cell source for obtaining the secretome. Further functional investigations are currently conducted to identify the most beneficial culturing conditions for obtaining prASC secretomes with positive therapeutic outcomes.

## Electronic Supplementary Material

Below is the link to the electronic supplementary material.

Supplementary Material 1

Supplementary Material 2

Supplementary Material 3

## Data Availability

Further information and requests for resources and reagents should be directed to and will be fulfilled by the corresponding author, Alexandra M. Smink (a.m.smink@umcg.nl).
