# Supplementary material for: Culturing Conditions Dictate the Composition and Pathways Enrichment of Human and Rat Perirenal Adipose-Derived Stromal Cells’ Secretomes
Source: Stem Cell Rev Rep. 2024 Jun 26;20(7):1869–88. doi: 10.1007/s12015-024-10748-w (PMC11445368; doi:10.1007/s12015-024-10748-w)
Supplement: Supplementary file 3 — Supplementary Material 3 [file 12015_2024_10748_MOESM3_ESM.docx]

**Author contributions**

Conceptualization, E.P.M., A.M.S.; Methodology, E.P.M., C.M., B.J.H., A.M.S.; Formal analysis, E.P.M.; Investigation, E.P.M.; Writing – Original Draft, E.P.M.; Writing – Review & Editing, E.P.M; M.M.F.; C.M., B.J.H.; A.M.S.; Funding Acquisition, A.M.S; Supervision, M.M.F. and A.M.S.; Project Administration, A.M.S.

**Declaration of interests**

The authors declare no competing interests.

**Resource availability**

Further information and requests for resources (e.g., Supplementary Files 1 and 2) and reagents should be directed to and will be fulfilled by the corresponding author, Alexandra M. Smink ([a.m.smink@umcg.nl](mailto:a.m.smink@umcg.nl)).

**SUPPLEMENTAL INFORMATION**

**
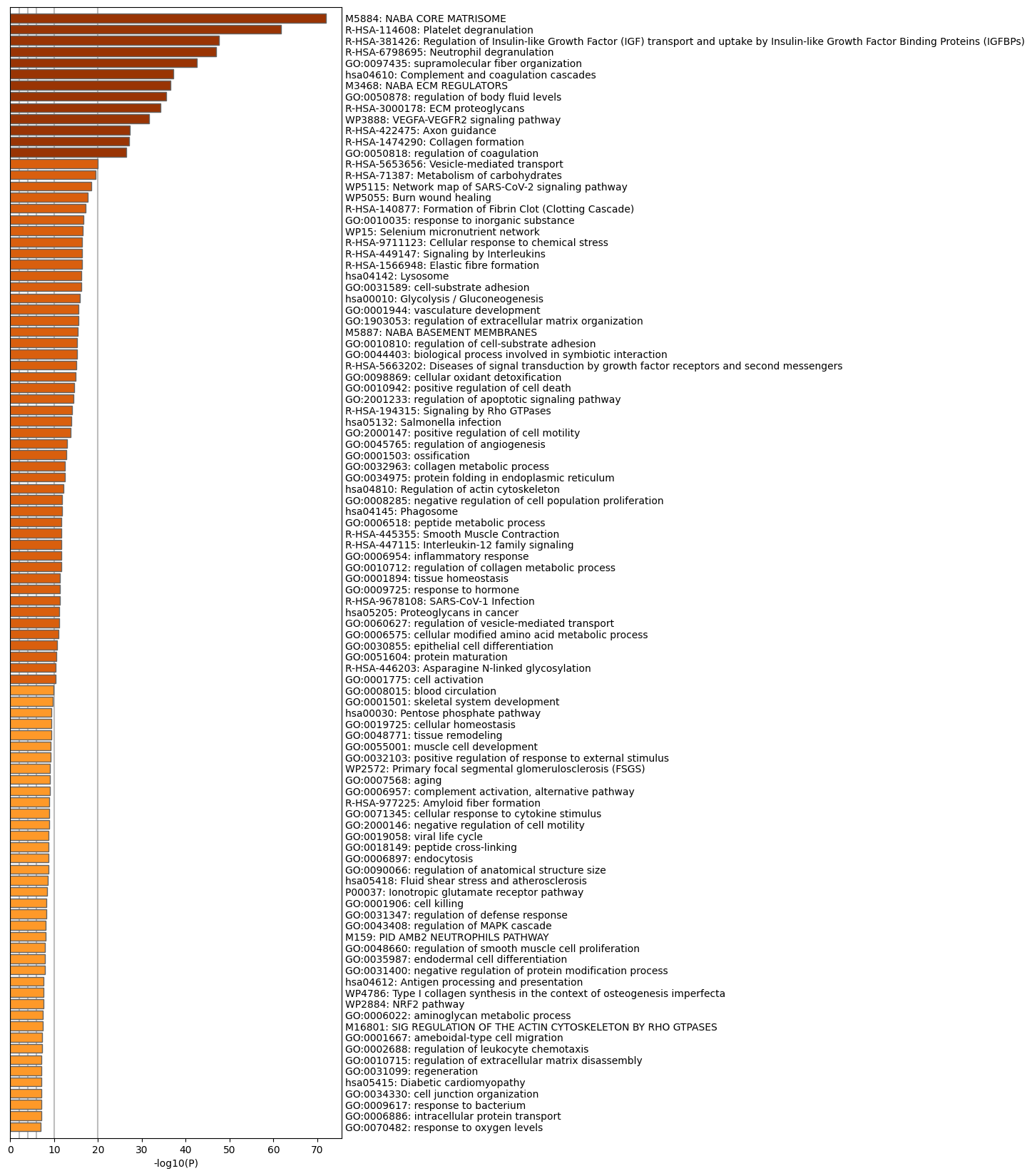
**

**Figure S1.** h-prASC secretome resulted of normoxia exposure. Metascape bar graphs for viewing top non-redundant enrichment clusters, using a colour scale to represent statistical significance.


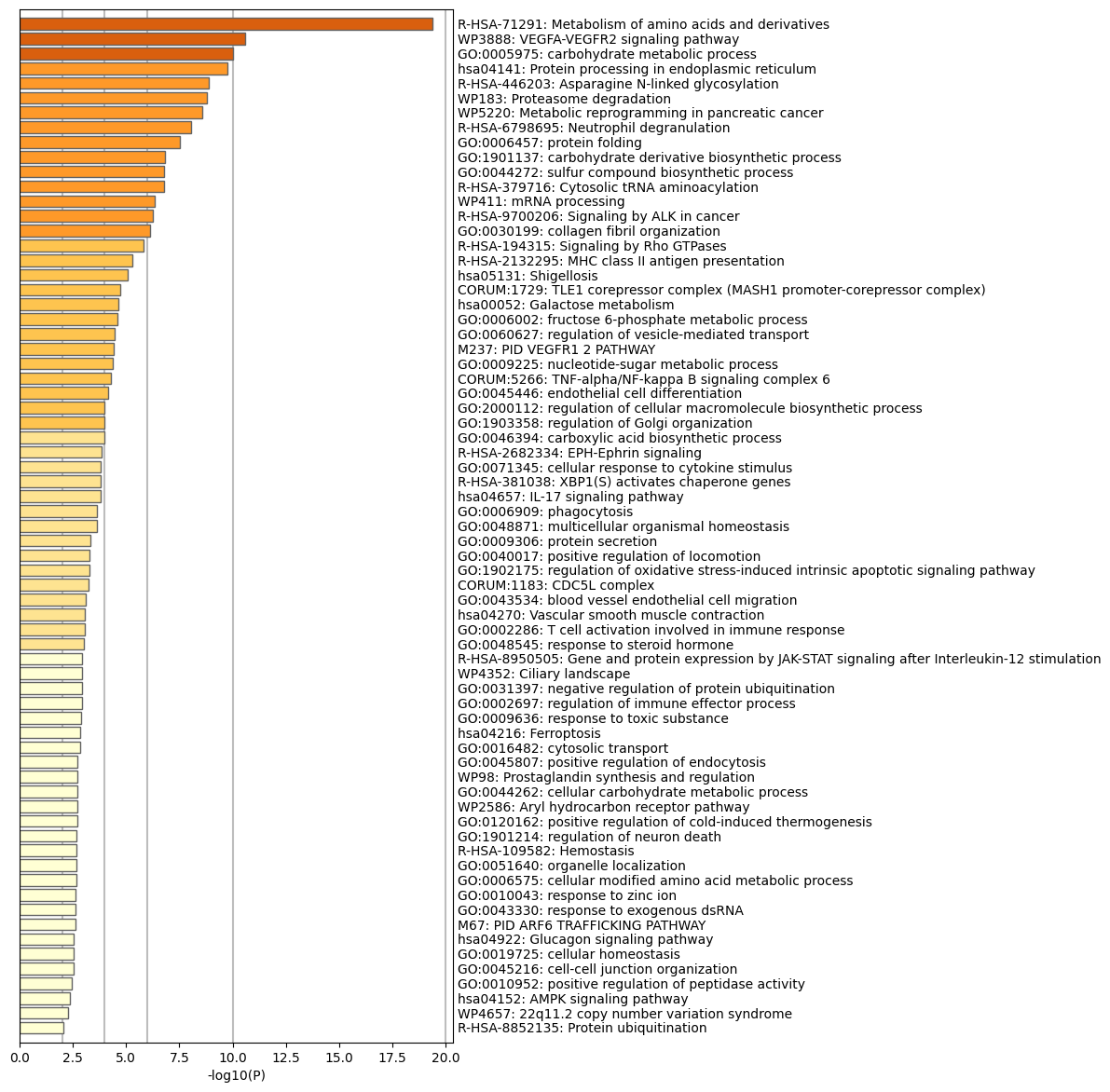


**Figure S2.** h-prASC secretome resulted of cytokines exposure. Metascape bar graphs for viewing top non-redundant enrichment clusters, using a colour scale to represent statistical significance.


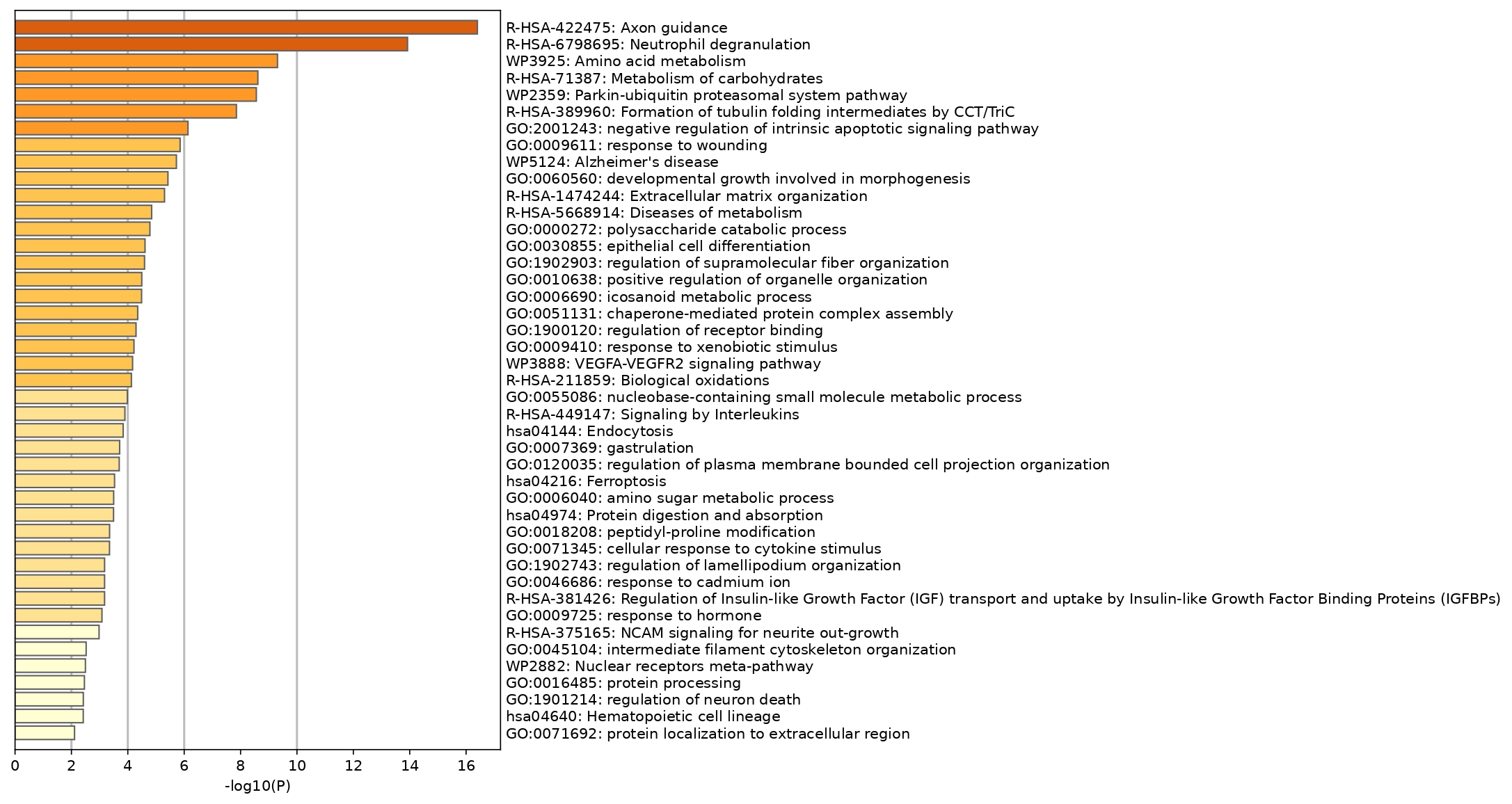
**Figure S3.** h-prASC secretome resulted of high glucose exposure. Metascape bar graphs for viewing top non-redundant enrichment clusters, using a colour scale to represent statistical significance.


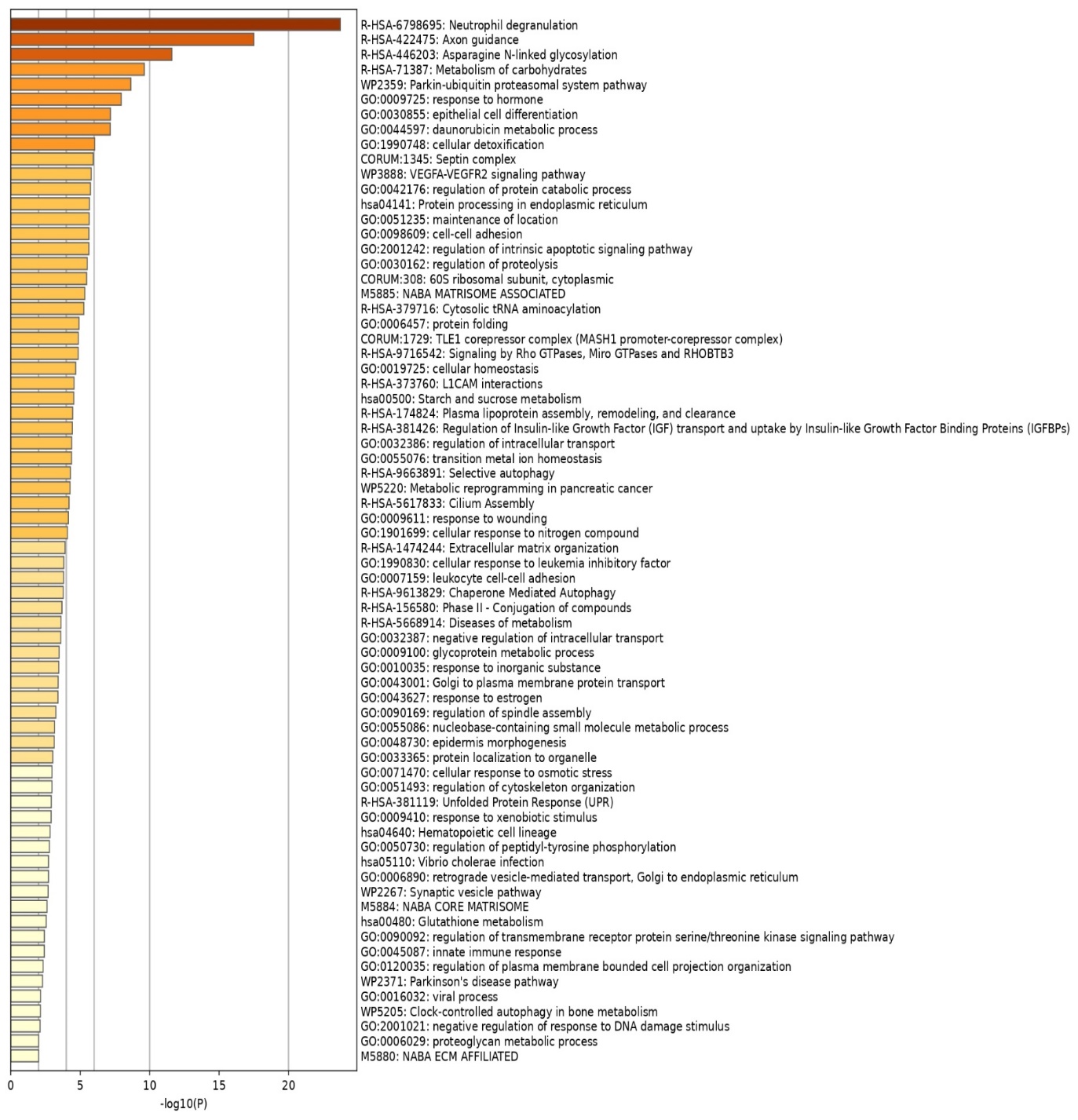
**Figure S4.** h-prASC secretome resulted of hypoxia exposure. Metascape bar graphs for viewing top non-redundant enrichment clusters, using a colour scale to represent statistical significance.


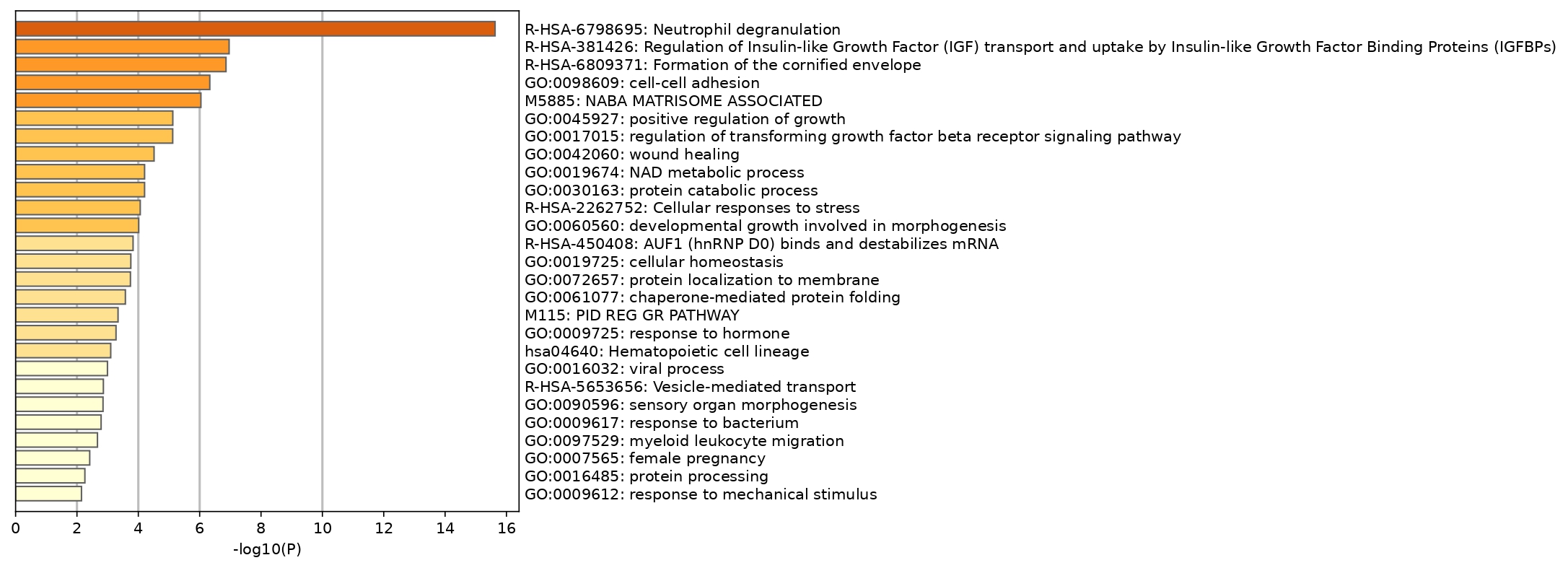
**Figure S5.** h-prASC secretome resulted of hypoxia and high glucose exposure. Metascape bar graphs for viewing top non-redundant enrichment clusters, using a colour scale to represent statistical significance


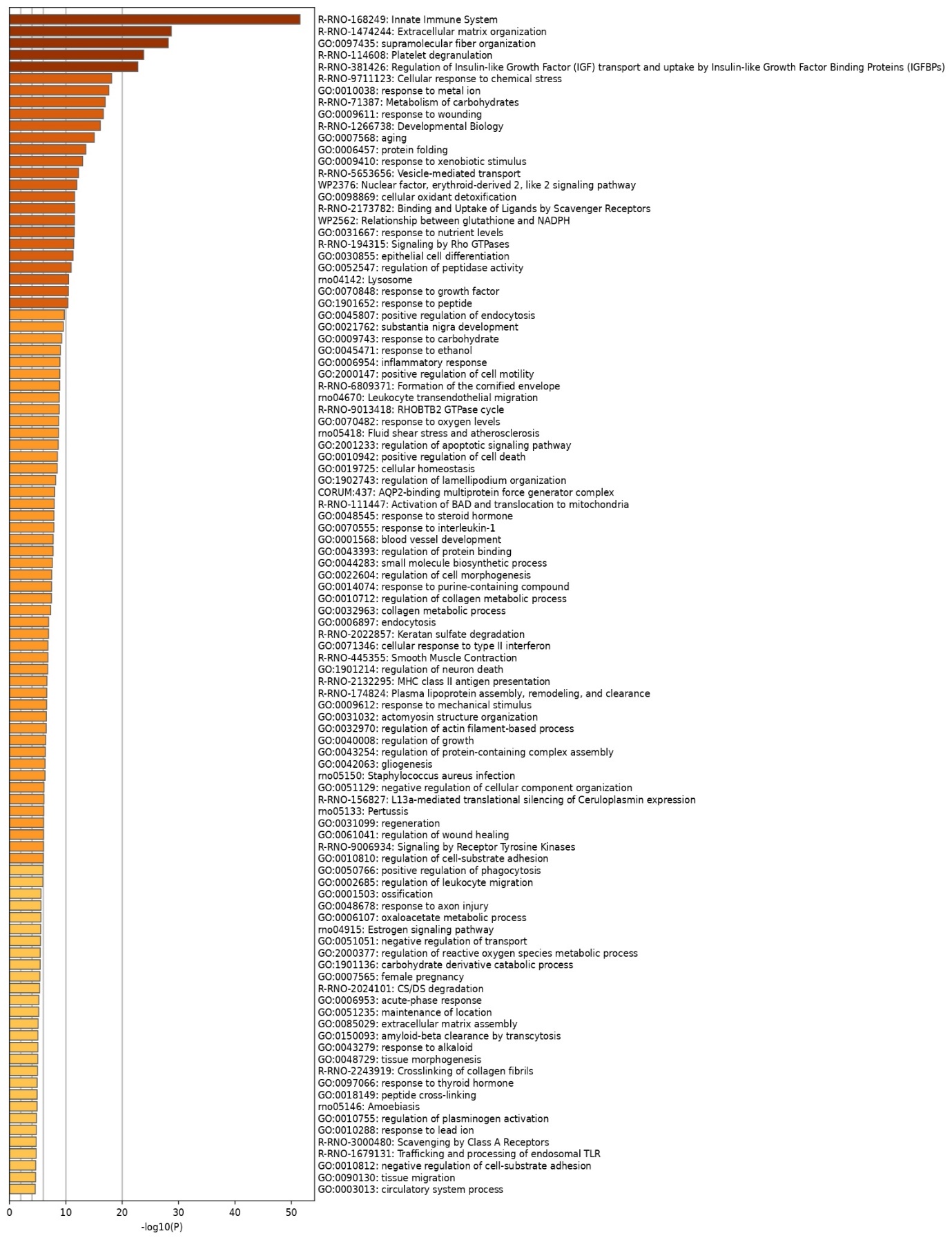
**Figure S6.**  top non-redundant enrichment clusters, using a colour scale to represent statistical significance. Metascape bar graphs for viewing top non-redundant enrichment clusters, using a colour scale to represent statistical significance.


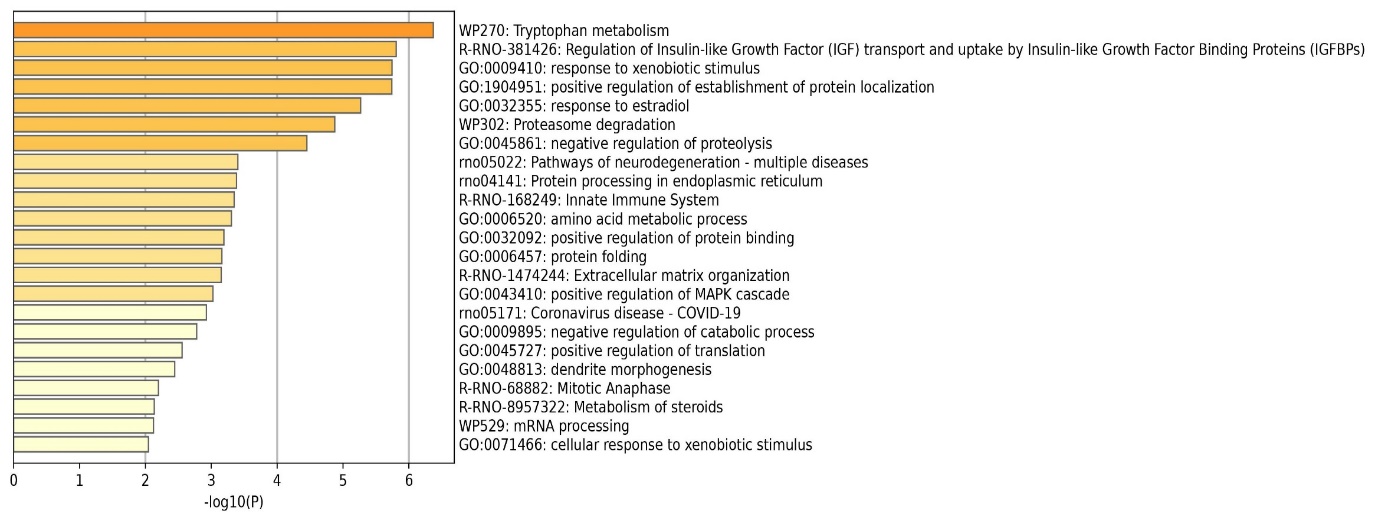
**Figure S7.** r-prASC secretome resulted of cytokines exposure. Metascape bar graphs for viewing top non-redundant enrichment clusters, using a colour scale to represent statistical significance.


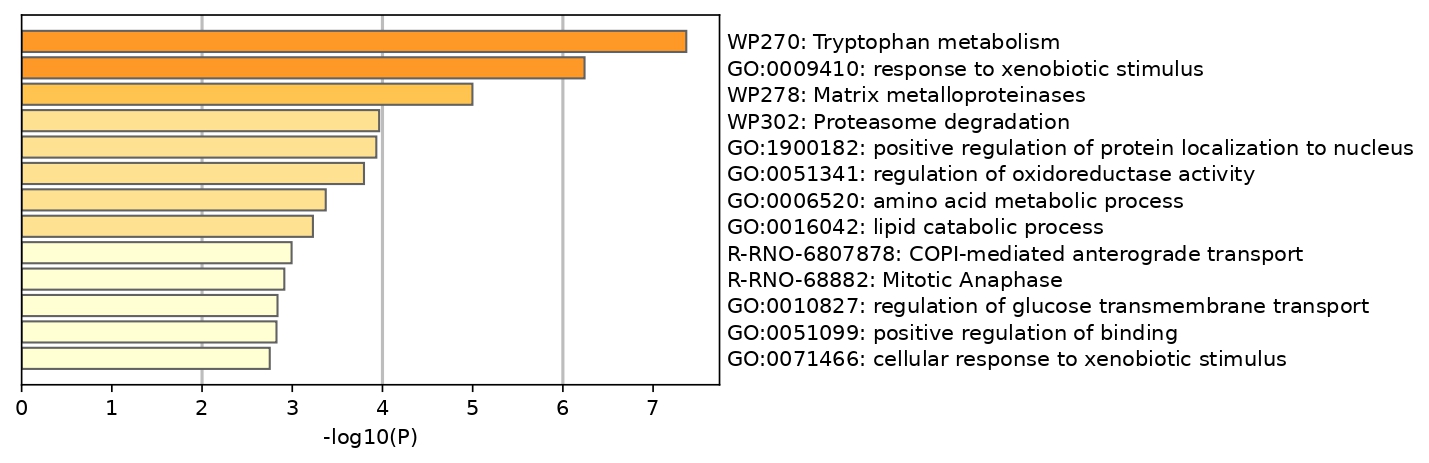


**Figure S8.**  r-prASC secretome resulted of high glucose exposure. Metascape bar graphs for viewing top non-redundant enrichment clusters, using a colour scale to represent statistical significance.


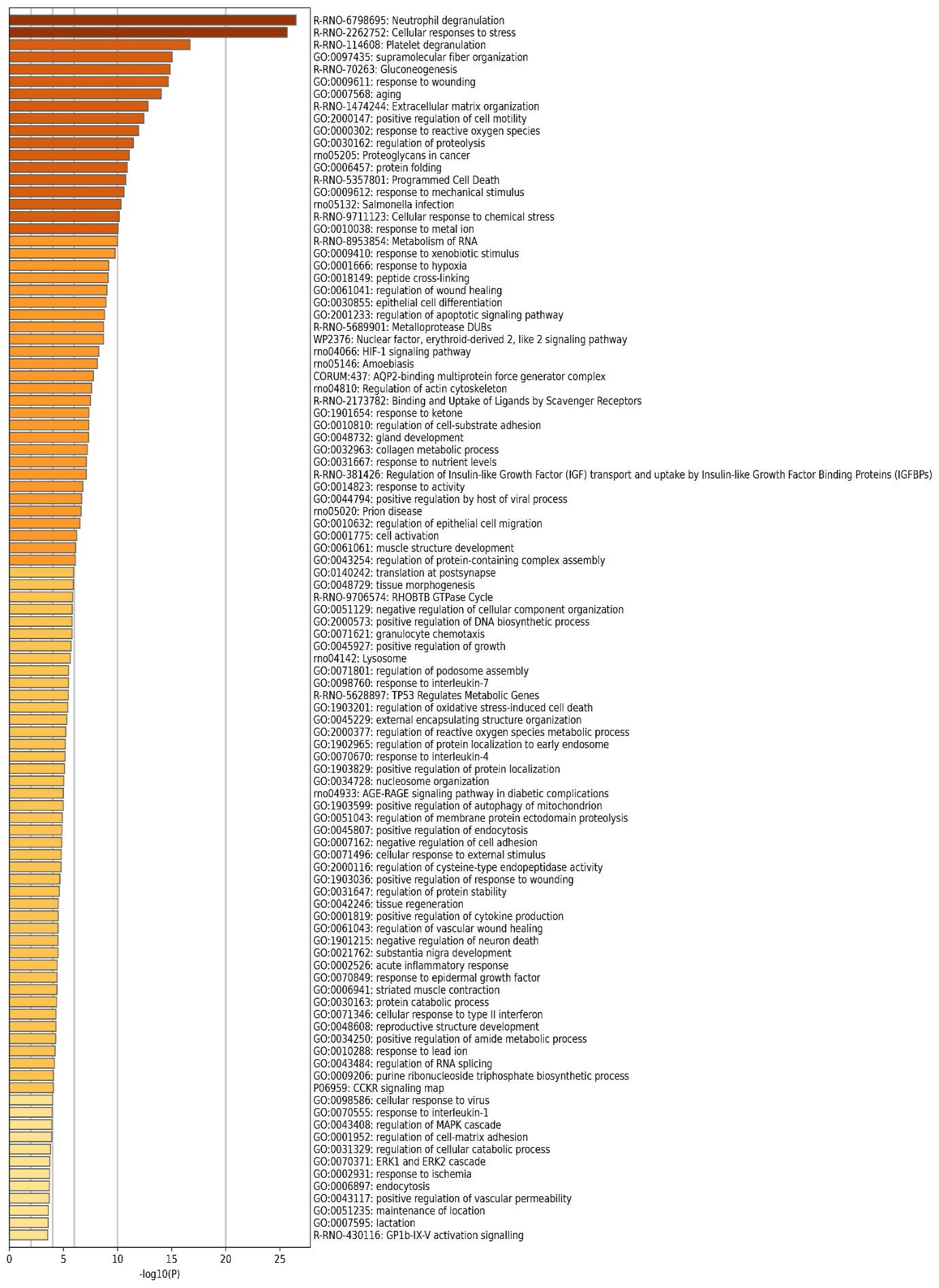
**Figure S9.** r-prASC secretome resulted of hypoxia exposure. Metascape bar graphs for viewing top non-redundant enrichment clusters, using a colour scale to represent statistical significance.


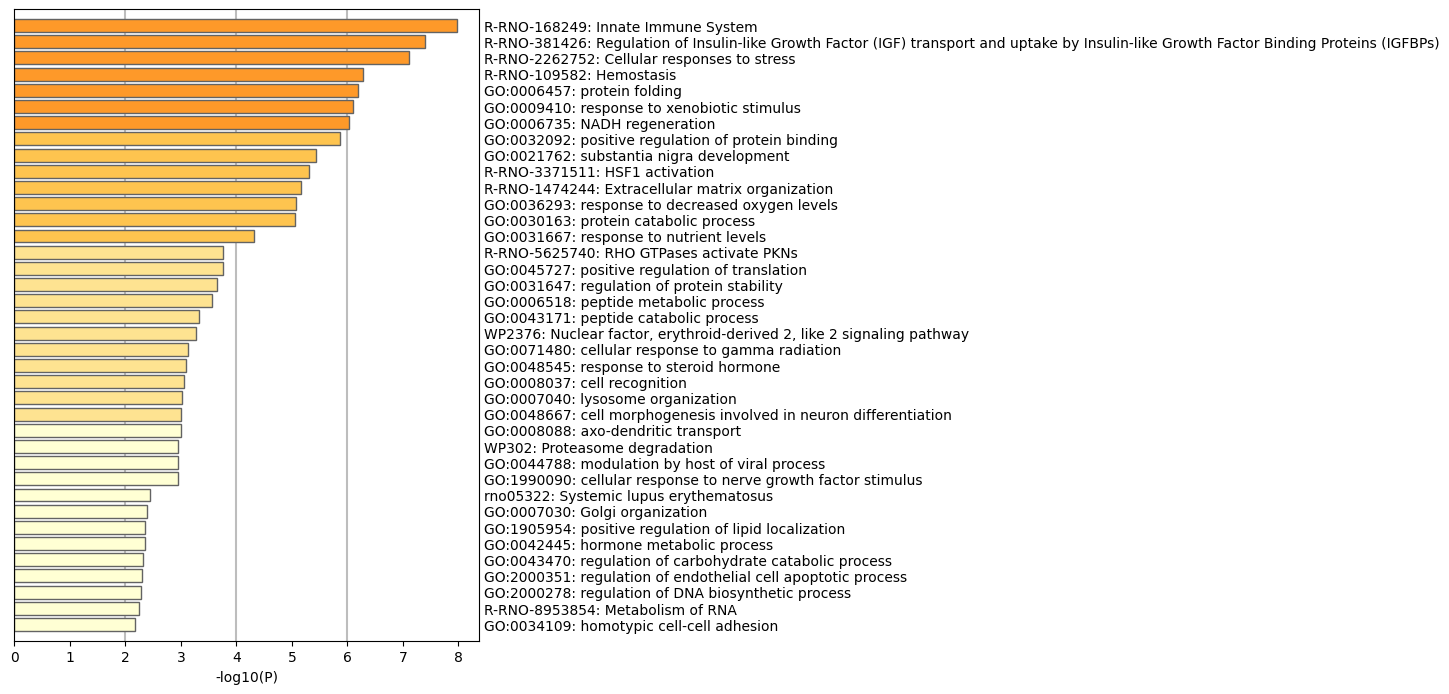
**Figure S10.** r-prASC secretome resulted of hypoxia and high glucose exposure. Metascape bar graphs for viewing top non-redundant enrichment clusters, using a colour scale to represent statistical significance.
